# BET bromodomain inhibition potentiates radiosensitivity in models of H3K27-altered diffuse midline glioma

**DOI:** 10.1172/JCI174794

**Published:** 2024-05-21

**Authors:** Jun Watanabe, Matthew R. Clutter, Michael J. Gullette, Takahiro Sasaki, Eita Uchida, Savneet Kaur, Yan Mo, Kouki Abe, Yukitomo Ishi, Nozomu Takata, Manabu Natsumeda, Samantha Gadd, Zhiguo Zhang, Oren J. Becher, Rintaro Hashizume

**Affiliations:** 1 Department of Pediatrics, Northwestern University Feinberg School of Medicine, Chicago, Illinois, USA.; 2 Division of Hematology, Oncology, Neuro-Oncology and Stem Cell Transplantation, Ann & Robert H. Lurie Children’s Hospital of Chicago, Chicago, Illinois, USA.; 3 Department of Pediatrics, University of Alabama at Birmingham, Birmingham, Alabama, USA.; 4 Department of Neurological Surgery, Brain Research Institute, Niigata University, Niigata, Japan.; 5 Department of Molecular Biosciences,; 6 High Throughput Analysis Laboratory, and; 7 Department of Neurological Surgery, Northwestern University Feinberg School of Medicine, Chicago, Illinois, USA.; 8 Department of Neurological Surgery, Wakayama Medical University, Wakayama, Japan.; 9 Institute for Cancer Genetics,; 10 Department of Pediatrics, and; 11 Department of Genetics and Development, Columbia University Irving Medical Center, New York, New York, USA.; 12 Center for Vascular and Developmental Biology, Feinberg Cardiovascular and Renal Research Institute, and; 13 Simpson Querrey Institute for BioNanotechnology, Northwestern University Feinberg School of Medicine, Chicago, Illinois, USA.; 14 Department of Pediatrics, Icahn School of Medicine at Mount Sinai, New York, New York, USA.; 15 Division of Pediatric Hematology and Oncology, Children’s of Alabama, Birmingham, Alabama, USA.; 16 O’Neal Comprehensive Cancer Center, University of Alabama at Birmingham, Birmingham, Alabama, USA.

**Keywords:** Oncology, Therapeutics, Brain cancer, Epigenetics, Translation

## Abstract

Diffuse midline glioma (DMG) H3K27-altered is one of the most malignant childhood cancers. Radiation therapy remains the only effective treatment yet provides a 5-year survival rate of only 1%. Several clinical trials have attempted to enhance radiation antitumor activity using radiosensitizing agents, although none have been successful. Given this, there is a critical need for identifying effective therapeutics to enhance radiation sensitivity for the treatment of DMG. Using high-throughput radiosensitivity screening, we identified bromo- and extraterminal domain (BET) protein inhibitors as potent radiosensitizers in DMG cells. Genetic and pharmacologic inhibition of BET bromodomain activity reduced DMG cell proliferation and enhanced radiation-induced DNA damage by inhibiting DNA repair pathways. RNA-Seq and the CUT&RUN (cleavage under targets and release using nuclease) analysis showed that BET bromodomain inhibitors regulated the expression of DNA repair genes mediated by H3K27 acetylation at enhancers. BET bromodomain inhibitors enhanced DMG radiation response in patient-derived xenografts as well as genetically engineered mouse models. Together, our results highlight BET bromodomain inhibitors as potential radiosensitizer and provide a rationale for developing combination therapy with radiation for the treatment of DMG.

## Abstract 2

BET bromodomain inhibition enhances the radiation response in diffuse midline glioma via downregulation of DNA repair gene expression–associated H3K27acetylation occupancy at enhancer regions.

## Graphical abstract

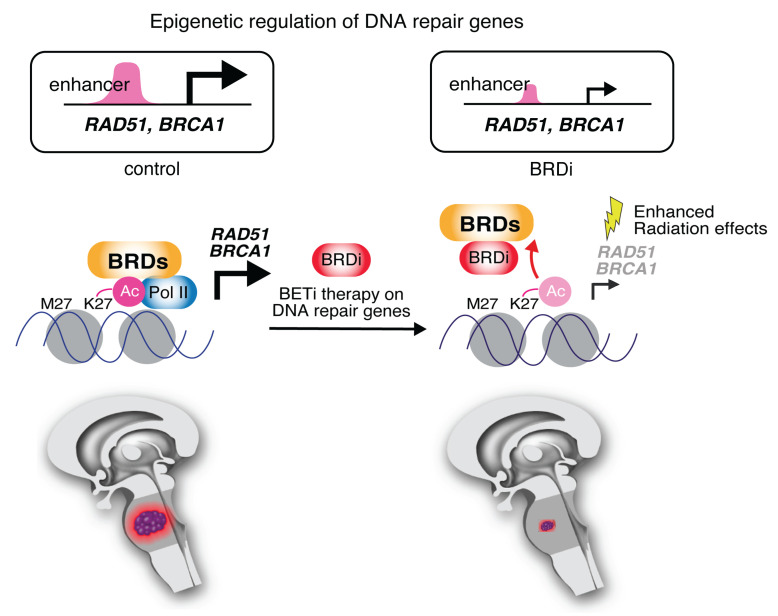

## Introduction

Diffuse midline gliomas (DMGs) with H3K27M mutation (histone H3 lysine 27 replaced with methionine) are diffusely infiltrating glial neoplasms affecting midline structures of the CNS (1). DMG is one of the most malignant childhood tumors, with a median survival of 9–12 months from diagnosis (2). Factors that contribute to the dismal prognosis include the infiltrative nature and anatomic location of the tumor within the pons, which precludes surgical resection. The identification of effective therapies has been extremely challenging, with more than 250 clinical trials involving different combinations of chemotherapeutic agents commonly used in adult glioma proving ineffective in treating DMG (3). Fractionated focal radiation to a total dose of 54–60 Gy over a 6-week period remains the only standard treatment modality that can provide transient symptom relief and a delay in tumor progression in about 70%–80% of patients. However, radiation-treated children with DMG show evidence of disease progression within the first year of completing radiation therapy (4, 5). Given this reality, the identification of efficacious therapeutic agents that enhance the antitumor effects of radiation is urgently needed for improving treatment outcomes for this patient population.

In contrast to adult gliomas, DMG is uniquely dependent on the H3K27M mutation for its initiation and maintenance (6–9). H3K27M mutation occurs in *H3F3A* and *HIST1H3B/C* genes, encoding histone H3 variants H3.3 and H3.1, respectively, in as much as 80% of DMGs and is associated with shorter survival among patients with DMG (6, 7, 10). We and others have identified a key functional consequence of H3K27M mutation: mutant protein sequestration of the polycomb repressive complex 2 (PRC2) methyltransferase resulting in functional inactivation of PRC2 (8, 9, 11, 12). This inactivation leads to a global reduction of H3K27 di-methylation (K27me2) and tri-methylation (K27me3), which, in turn, leads to extensive transcriptional reprogramming of mutant cells and promotes a stem cell–like, therapy-resistant phenotype.

While decreasing H3K27 methylation, K27M mutation also increases K27 acetylation (K27ac), which is necessary for bromo- and extraterminal domain (BET) protein transcriptional activation through RNA polymerase II (13–15). We have previously profiled the epigenome of H3K27M-mutant DMG cells and found that K27M mutant colocalized with K27ac (15). Heterotypic H3K27M-K27ac nucleosomes colocalized with BET bromodomain proteins 2 and 4 (BRD2 and BRD4) at actively transcribed gene loci to activate transcription in DMG (15). Highly selective BET bromodomain inhibitors, such as JQ1 and I-BET (16–19), suppress gene transcription by blocking binding between BRD and acetylated histones, representing a promising therapeutic strategy for treating DMG (13–15, 20). Indeed, targeted BET bromodomain activity using JQ1 inhibited tumor growth and extended the survival of animals bearing H3K27M-mutant DMG patient–derived xenografts (PDXs) (15). Because of their promising antitumor activity, BET bromodomain inhibitors are being tested in several clinical trials with cancer patients including those with myeloma (ClinicalTrials.gov NCT03068351), leukemia (NCT02158858), prostate cancer (NCT02711956), and other advanced solid cancers (NCT01587703, NCT02419417), including pediatric brain cancer (NCT03936465). However, how small molecule inhibitors, such as those targeting BET bromodomain activity, interact with radiation has not to our knowledge been explored in DMG.

Through an unbiased high-throughput radiosensitivity screen, we found the BET bromodomain inhibitors to be potent radiosensitizers of H3K27M-mutant DMG cells. The depletion of BRD using shRNAs and sgRNAs, and BET bromodomain inhibition using small molecule inhibitors, reduced DMG cell proliferation and enhanced radiation-induced DNA damage by inhibiting DNA repair pathways. Moreover, BET bromodomain inhibition downregulated expression of DNA repair genes associated with H3K27 acyetylation (H3K27ac) occupancy and enhanced DMG radiation response in vitro and in vivo. Together, these results highlight BET bromodomain inhibition as a potential radiosensitizer and provide a rationale for developing combination therapy with radiation for the treatment of this deadly pediatric brain cancer.

## Results

### BET bromodomain inhibitors are identified as potent radiosensitizers by high-throughput drug screening.

**Figure 1:**
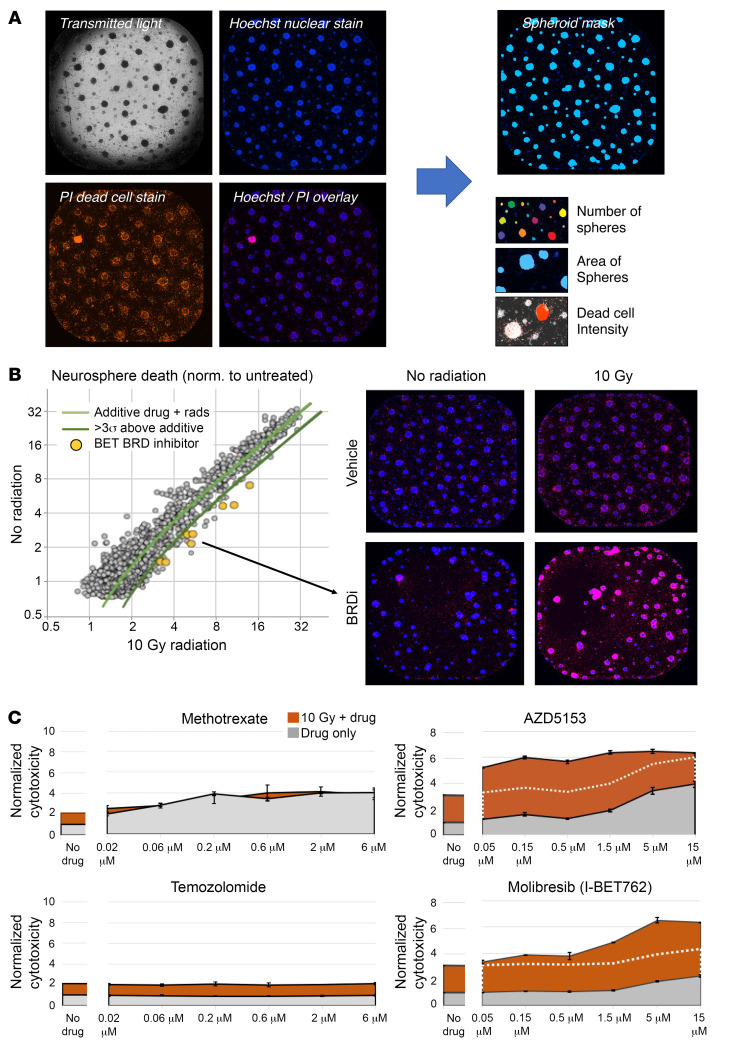
High-throughput drug screening with radiation identified BET bromodomain inhibitors as radiosensitizers in DMG cells. Tumor cells isolated from GEMM-DMG (Ntv-a; p53fl/fl; PDGFB; H3.3K27M; Cre) mice were cultured ex vivo as neurospheres and used to drug screen for radiosensitizers. (**A**) Representative image of neurospheres in transmitted light, Hoechst staining (nuclear), PI staining (dead cell), and Hoechst/PI overlay. Evaluation of the number, area, and dead cell intensity of neurospheres. 4x magnification, with each pixel being 3.4156 mm in size, for a raw image of 3.5 mm. Images were subsequently enlarged to enhance visibility. (**B**) A library of 1,280 FDA-approved drugs and 1,600 clinical candidates was screened in the presence or absence of 10 Gy radiation. Left: Compounds to the right of the diagonal light green line are identified as having an additive effect on neurospheres in the presence 10 Gy radiation Compounds to the right of the dark green diagonal line are identified as those that radiosensitized neurospheres beyond the additive drug effect when combined with 10 Gy radiation (at ≥3σ). Right: Representative images of neurospheres treated with BET bromodomain inhibitor (BRDi) in the presence or absence of 10 Gy radiation. norm., normalized. 4× magnification, with each pixel being 3.4156 mm in size, for a raw image of 3.5 mm. Images were subsequently enlarged to enhance visibility. (**C**) Radiosensitizing effect (orange) and cytotoxic effect (gray) with methotrexate, AZD5153, temozolomide, and molibresib (I-BET762).

We first performed an unbiased high-throughput radiosensitivity screen in H3.3 WT and K27M-mutant DMG neurosphere cells using a total of 2,880 compounds, including 1,280 FDA-approved drugs and 1,600 clinical compounds (mainly small molecule inhibitors of epigenetic processes) in the presence or absence of 10 Gy irradiation. Radiosensitizing effects were quantified by cell death number using confocal image analysis combined with Hoechst nuclear staining and propidium iodide (PI) DNA staining (Figure 1A). We identified several clinical-grade BET bromodomain inhibitors as potent radiosensitizers in the screen (Supplemental Table 1; supplemental material available online with this article; https://doi.org/10.1172/JCI174794DS1), which increased cell death in combination with radiation (Figure 1B and Supplemental Figure 1). H3.3 K27M-mutant DMG neurosphere cells were more sensitive to BET bromodomain inhibitors in combination with radiation than H3.3 WT DMG neurosphere cells (Supplemental Figure 1). These BET bromodomain inhibitors were subsequently validated for their antiproliferative effects in DMG cells. AZD5153 and molibresib (I-BET762) in combination with radiation showed strong additive cytotoxic effects relative to each monotherapy (white dotted line, Figure 1C). However, methotrexate and temozolomide, which have been using in combination with radiation in adult glioblastoma (GBM), did not show additive radiosensitizing effects or monotherapy cytotoxic effects in DMG cells. Our results are consistent with the results from clinical trials, which show that DMG transiently responds to the combination of temozolomide and radiation, but with no survival benefit from the combination therapy (21).

### Targeted inhibition of BET bromodomain activity reduces cell proliferation and induces apoptosis in K27M-mutant DMG cells.

**Figure 2:**
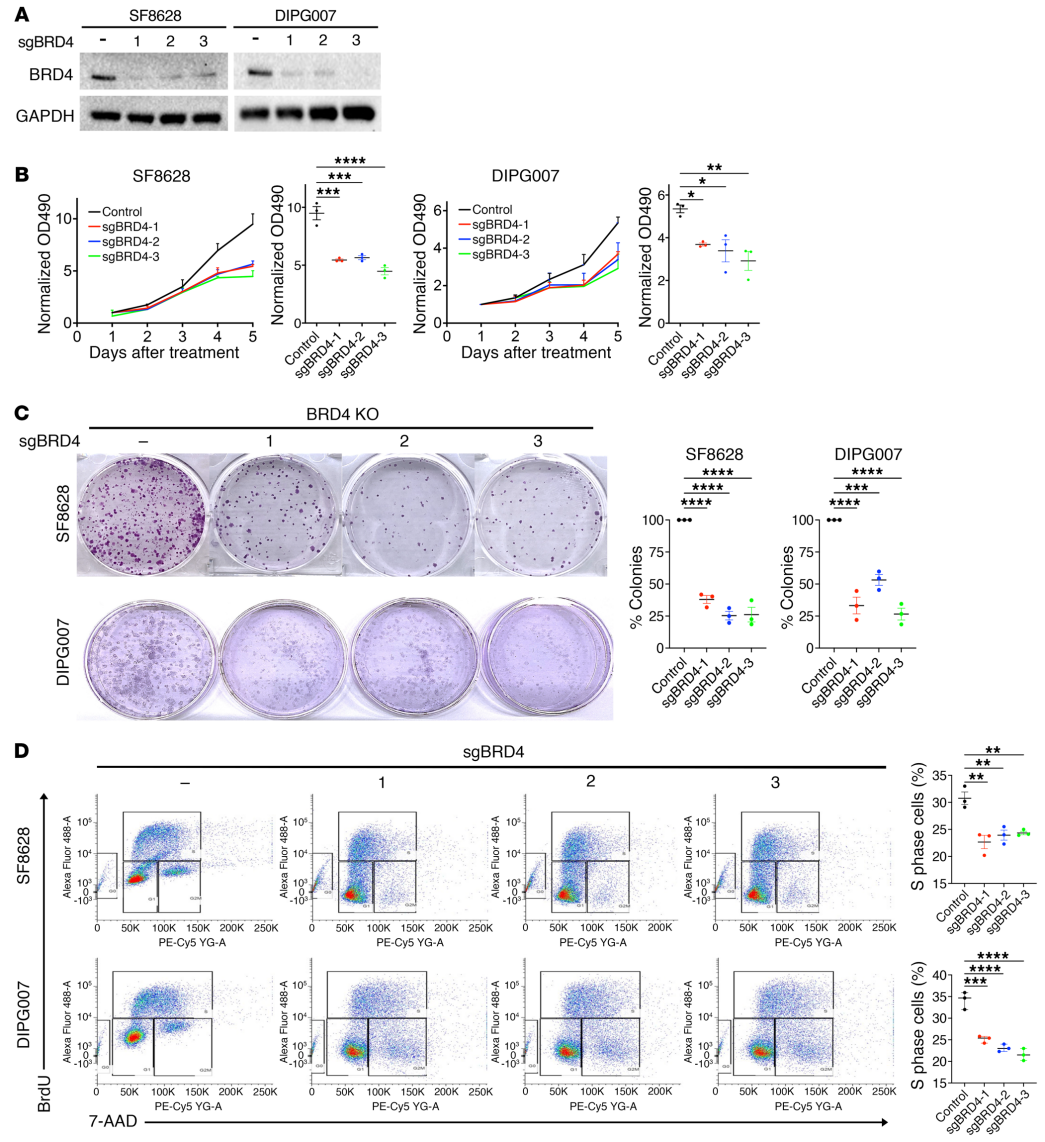
BRD4 depletion suppressed cell growth in DMG cells. (**A**) Western blotting results showing sgRNA-mediated depletion of BRD4 expression (sgBRD4-1, -2, -3) in SF8628 and DIPG007 cells. (**B**) Cell growth plot showing antiproliferative effects of sgBRD4-1, -2, and -3 in SF8628 and DIPG007 cells. The plot represents the absorbance (OD, λ = 490 nm) quantified each day (left). Values shown are the average (mean ± SEM) from triplicate samples for each condition as day 1 normalized. Dot plot representation of OD_490_ values on day 5 (right). Statistical analysis was performed using 1-way ANOVA comparisons: SF8628, sgBRD4-1, ***P = 0.0001; sgBRD4-2, ***P = 0.0002; sgBRD4-3, ****P < 0.0001; DIPG007, sgBRD4-1, *P = 0.0261; sgBRD4-2, *P = 0.0113; sgBRD4-3, **P = 0.0033. *n* = 3 (**C**) Effect of BRD4 depletion in colony formation. Bar graphs: Representation of colony numbers in DMG cells. Values shown are the average (mean ± SEM) from triplicate samples for each condition. One-way ANOVA comparisons between the control and BRD4 depletion groups, ****P < 0.0001; sgBRD4-2, ***P = 0.0004. (**D**) Effects of BRD4 depletion in BrdU incorporation assay. Cells were pulsed with 10μM BrdU for 1 hour and treated with Alexa Fluor 488–BrdU antibody and 7-aminoactinomycin D (7-AAD). Bar graphs represent BrdU-positive cell numbers. Values shown are the average (mean ± SEM) from duplicate samples for each incubation condition. S-phase cell populations were analyzed with 1-way ANOVA comparisons of each BRD4 sgRNA, ****P < 0.0001; SF8628: sgBRD4-1, **P = 0.0017; sgBRD4-2, **P = 0.0050; sgRNA3 **P = 0.0072; DIPG007: sgBRD4-1, ***P = 0.0001.

To address whether BET bromodomain activity is required for K27M-mutant DMG cell growth, we studied the effects of depletion of BRDs (BRD2, -3, -4) on DMG cell proliferation using CRISPR/Cas9 KO (Supplemental Figure 2). KO effects were confirmed at the protein level (Supplemental Figure 2A), and the effects of BRD depletion on cell proliferation were analyzed by use of the MTS assay (Supplemental Figure 2B). BRD4 depletion reduced H3K27ac and reciprocally increased H3K27me3 protein expression, whereas depletion of BRD2 and BRD3 did not affect the expression levels of H3K27ac and K27me3 (Supplemental Figure 2A). In addition, only BRD4 depletion suppressed the growth of DMG cells (Supplemental Figure 2B). We further analyzed the effects of BRD4 depletion on DMG cell growth (Figure 2 and Supplemental Figure 3). BRD4 KO and shRNA knockdown (KD) were confirmed at the protein level (Figure 2A and Supplemental Figure 3A), and the effects of BRD4 depletion on cell proliferation were analyzed by the MTS assay (Figure 2B and Supplemental Figure 3B), colony formation assays (Figure 2C and Supplemental Figure 3C), and BrdU incorporation assay (Figure 2D) in 2 K27M-mutant DMG cell lines (SF8628 and DIPG007). BRD4 depletion significantly reduced DMG cell growth relative to scramble control (Figure 2B and Supplemental Figure 3B). BRD4 depletion also suppressed colony formation in DMG cells (Figure 2C and Supplemental Figure 3C). The BrdU-positive cell population in S phase was decreased by BRD4 depletion in DMG cells (Figure 2D). These results indicate that BRD4 activity is required for DMG cell proliferation and suggest the possibility of a rational therapeutic target in DMG.

**Figure 3:**
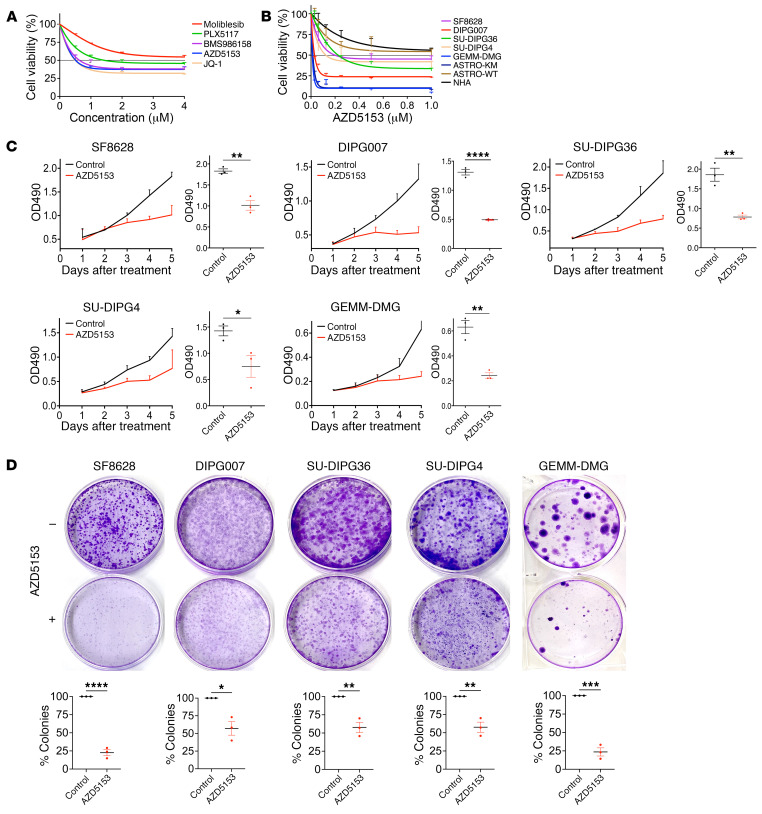
BET bromodomain inhibitors suppressed cell growth in DMG cells. (**A**) Cell growth plot showing antiproliferative effects of clinical-grade BET bromodomain inhibitors (molibresib, PLX5117, BMS986158, AZD5153, JQ-1) at 0–4 μM in SF8628 cells. Values shown are the average (mean ± SD) from triplicate samples for each incubation condition. (**B**) Proliferation response of DMG cells (SF8628, DIPG007, SU-DIPG36, SU-DIPG4, GEMM-DMG) and Astro-KM, Astro-WT, and NHA cells to increasing concentration of AZD5153. Values shown are the average (mean ± SEM) from triplicate samples for each incubation condition. (**C**) Cell growth plot showing proliferation response to IC_50_ values of AZD5153 of SF8628, DIPG007, SU-DIPG36, SU-DIPG4, and GEMM-DMG cells at each time point. The plot represents an OD value at 490 nm. Values shown are the average (mean ± SEM) from triplicate samples for each condition. Dot plot representation of OD_490_ values on day 5. Statistical analysis was performed using a 2-tailed unpaired *t* test: SF8628, **P = 0.0029; DIPG007, ****P < 0.0001; SU-DIPG36, **P = 0.0034; SU-DIPG4, *P = 0.0040; GEMM-DMG, **P = 0.0025. (**D**) Colony-forming effect on cells treated with IC_50_ values of AZD5153. Bar graph representation of colony numbers in the DMG cells treated with DMSO (0.5%) or IC_50_ values of AZD5153. Values shown are the average (mean ± SEM) from triplicate samples for each condition. Unpaired *t* test values for comparisons between the absence and presence of AZD5153 (*n* = 3): SF8628, ****P < 0.0001; DIPG007, *P = 0.0109; SU-DIPG36, **P = 0.0038; SU-DIPG4, **P = 0.0037; GEMM-DMG, ***P = 0.0002.

We and others have shown that targeting BET bromodomain activity using the inhibitor JQ1 resulted in potent antitumor activity in patient-derived DMG cells and PDX models (15, 20). Despite its promising antitumor activity, JQ1 has not been translated to clinical trials due to its short half-life (16, 18, 22–25). Thus, we expanded our findings to test the efficacy of clinical-grade BET bromodomain inhibitors including molibresib (I-BET762), PLX51107, BMS-986158, and AZD5153 in SF8628 DMG cells (Figure 3A). Of those, AZD5153 and BMS-986158 showed potent growth inhibition of SF8628 DMG cell, with 50% growth inhibition (IC_50_) values of 0.41 μM and 0.69 μM, respectively, which were similar to the IC_50_ value for JQ1 (0.50 μM) (Figure 3A). AZD5153 and BMS-986158 have been used in phase 1 clinical trials for malignant solid tumors, including pediatric brain cancer and blood cancer (AZD5153: ClinicalTrials.gov NCT03205176, NCT03527147, NCT03013998; BMS-986158: NCT03936465, NCT04817007, NCT02419417, NCT05372354). However, the preclinical efficacy of BMS-986158 was disappointing in orthotopic (brainstem) DMG PDX models (Supplemental Figure 3D) due to its poor penetrance across the blood-brain barrier (BBB), with a brain penetration ratio of 5.02% ± 1.32 % (Supplemental Table 2). AZD5153 showed a brain penetration ratio of 12.9% ± 1.25 %, higher than that of BMS-986158. Thus, we tested the efficacy of AZD5153 for cytotoxicity and radiosensitivity in DMG in vitro and in vivo. AZD5153 treatments induced dose-dependent inhibition of cell growth in 5 K27M-mutant DMG cell lines as well as human astrocytes expressing the K27M *H3F3A* transgene (Astro-KM cells), with IC_50_ values of 0.41 μM (SF8628), 0.053 μM (DIPG007), 0.022 μM (SU-DIPG36), 0.063 μM (SU-DIPG4), 0.013 μM (genetically engineered mouse model–DMG [GEMM-DMG]), and 0.020 μM (Astro-KM) (Figure 3B). Normal human astrocytes (NHAs) and Astro-WT cells showed less sensitivity to AZD5153, with IC_50_ values of 1.35 μM and 1.47 μM, respectively (Figure 3B). AZD5153 IC_50_also inhibited DMG cell growth in a time-dependent manner (Figure 3C) and reduced colony formation in DMG cells (Figure 3D).

### BET bromodomain inhibition sensitizes DMG cells to radiation.

**Figure 4:**
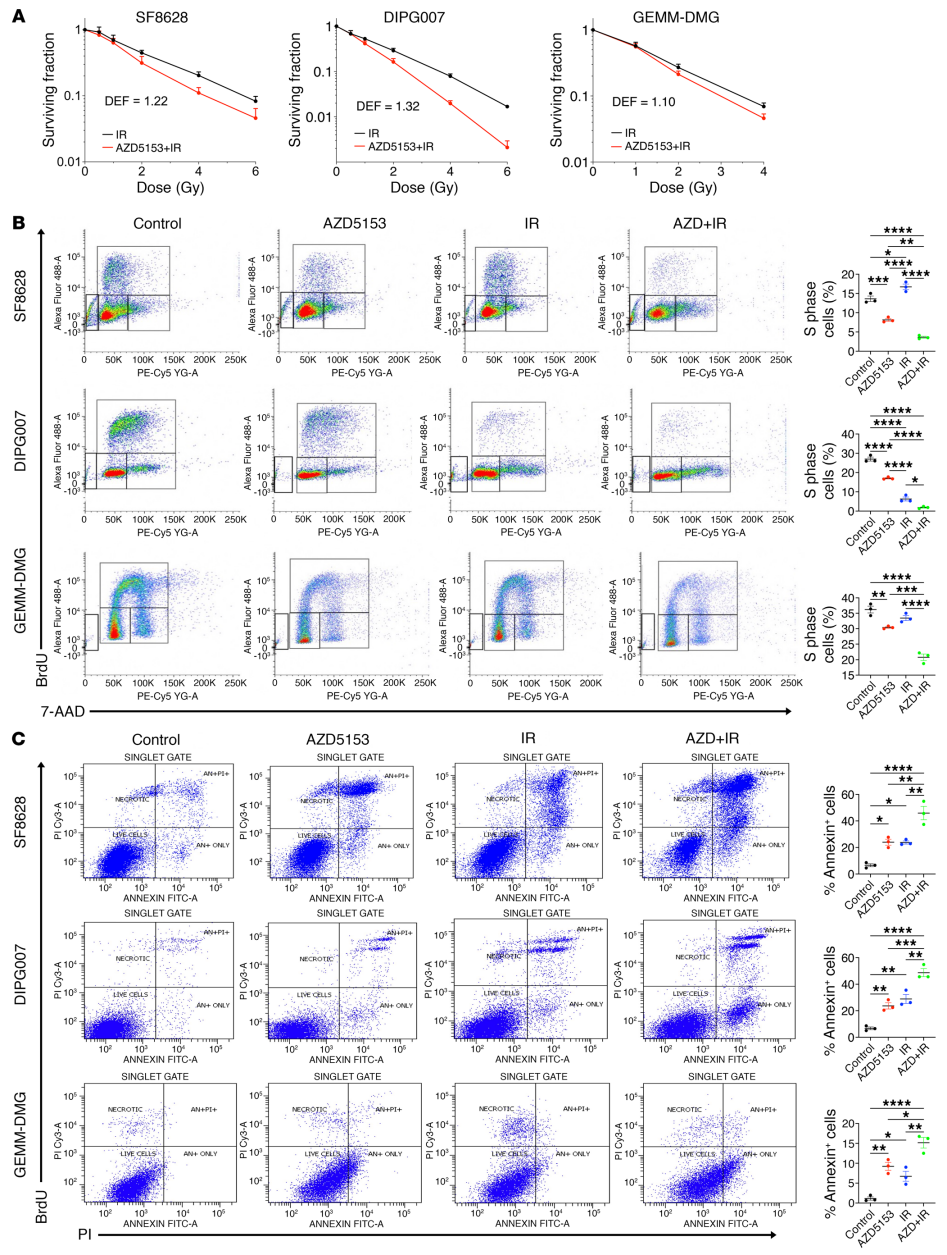
BET bromodomain inhibition increased radioresponse and apoptosis in DMG cells. (**A**) Clonogenic survival for K27M-mutant DMG cells (SF8628, DIPG007, GEMM-DMG) treated with AZD5153 (50 nM for SF8628 and DIPG007, 10 nM for GEMM-DMG) for 12 hours before being subjected to IR. Survival fractions, shown as mean ± SEM based on averages from triplicate samples, were normalized to plating efficiency. DEF was calculated at 10% survival level. (**B**) Effects of AZD5153 and IR on cell proliferation in BrdU incorporation assay. Cells were treated with 500 nM AZD5153 in the presence or absence of 4 Gy IR for 48 hours, pulsed with 10 μM BrdU for 1 hour, then analyzed by flow cytometry. Left: Cell sorting scatter plots for vehicle control– (0.5% DMSO), AZD5153-, and IR-treated cells are shown. Right: Graphs show S-phase composition. One-way ANOVA comparisons between each treatment (*n* = 3), ****P < 0.0001; control vs. AZD5153: ***P = 0.0005 (SF8628), **P = 0.0065 (GEMM-DMG); AZD5153 vs. AZD5153 + IR: **P = 0.0020 (SF8628), ***P = 0.0002 (GEMM-DMG); control vs. IR: *P = 0.0177 (SF8628), IR vs. AZD5153 + IR: *P = 0.0147 (DIPG007). (**C**) Annexin V analysis of AZD5153 apoptosis effects. Cells were treated with vehicle control (0.05% DMSO) or 1 μM AZD5153 concurrently with and without 6 Gy IR. Cells were collected after 48 hours and treated with Alexa Fluor 488–Annexin V and flow sorted. Bar graphs represent Annexin V–positive cell numbers. One-way ANOVA comparisons of each treatment (*n* = 3), ****P < 0.0001; control vs. AZD5153: *P = 0.0115 (SF8628), **P = 0.0076 (DIPG007), **P = 0.0030 (GEMM-DMG); control vs IR: *P = 0.0124 (SF8628), **P = 0.0014 (DIPG007), *P = 0.0245 (GEMM-DMG); AZD5153 vs. AZD5153 + IR: **P = 0.0027 (SF8628), ***P = 0.0029 (DIPG007), *P = 0.0177 (GEMM-DMG); IR vs. AZD5153 + IR: **P = 0.0027 (SF8628), **P = 0.0029 (DIPG007), **P= 0.0022 (GEMM-DMG).

To verify the radiosensitizing effect of BET bromodomain inhibition in K27M-mutant DMG cells, we conducted a clonogenic survival assay in 3 DMG cell lines (SF8628, DIPG007, GEMM-DMG). Cells were treated with AZD5153 (Figure 4A) and JQ1 (Supplemental Figure 4A) and depleted of BRD4 with shBRD4 and sgBRD4 (Supplemental Figure 4B), concurrently with being subjected to ionizing radiation (IR). To quantify the radiosensitizing effect, we calculated the dose enhancement factor (DEF), which represents the ratio of the dose with IR alone to the dose with combination of BRD4 inhibitor plus IR and BRD4 inhibition at 10% survival. If the DEF is greater than 1, the BRD4 inhibition will be functioning as a radiosensitizer. AZD5153 treatment showed a radiation-enhancing effect, with DEFs of 1.22 (SF8628), 1.32 (DIPG007), and 1.10 (GEMM-DMG) (Figure 4A). JQ1 had similar effects on radiation response in DMG cells, with DEFs of 1.25 (SF8628), 1.40 (DIPG007), and 1.14 (GEMM-DMG) (Supplemental Figure 4A). shBRD4 KD and sgBRD4 KO also increased the radiation response of DMG cells, with DEFs of 1.22 (shBRD4-484 and -487, SF8628), 1.34 (shBRD4-484 and -487, DIPG007), and 1.18 (sgBRD4-1, SF8628), 1.20 (sgBRD4-2, SF8628), 1.28 (sgBRD4-1 and -2, DIPG007) (Supplemental Figure 4B). Furthermore, we conducted BrdU incorporation (Figure 4B), apoptosis (Figure 4C), senescence (Supplemental Figure 5, A and B), and sphere formation (Supplemental Figure 5, C and D) assays. AZD5153 treatment resulted in a decreased BrdU-positive S-phase cell population relative to control (Figure 4B). Combination treatment with AZD5153 plus IR further decreased the S-phase cell population when compared with AZD5153 alone (Figure 4B). The annexin V apoptosis assay showed that either AZD5153 or IR monotherapy increased the level of annexin-positive cells compared with control (Figure 4C). Combination treatment of AZD5153 plus IR increased the level of annexin V–positive cells, outperforming each monotherapy. The β-galactosidase assay revealed increasing senescence-associated β-galactosidase staining in DMG cells treated with either AZD5153 or IR monotherapy (Supplemental Figure 5A). Combination treatment with AZD5153 and IR further increased β-galactosidase–positive DMG cells. Cell size is known to be associated with senescence. To quantify cell size, we gated DMG cells for G_1_ DNA content and sorted them with the side scatter parameter (SCC) using flow cytometry (Supplemental Figure 5B). Similar to the results with β-galactosidase staining, combination treatment with AZD5153 and IR further increased cell size relative to either monotherapy. Combination treatment also reduced self-renewal activity (Supplemental Figure 5C) and neurosphere formation compared with either monotherapy (Supplemental Figure 5D). These results suggest that when compared with monotherapy, combination treatment with AZD5153 plus IR further increased radiosensitivity in DMG cells by decreasing the population of radioresistant S-phase cells and stemness and increasing apoptosis and senescence.

### BET bromodomain inhibition downregulates the genes involved in DNA repair and cell cycle in K27M-mutant DMG cells.

**Figure 5:**
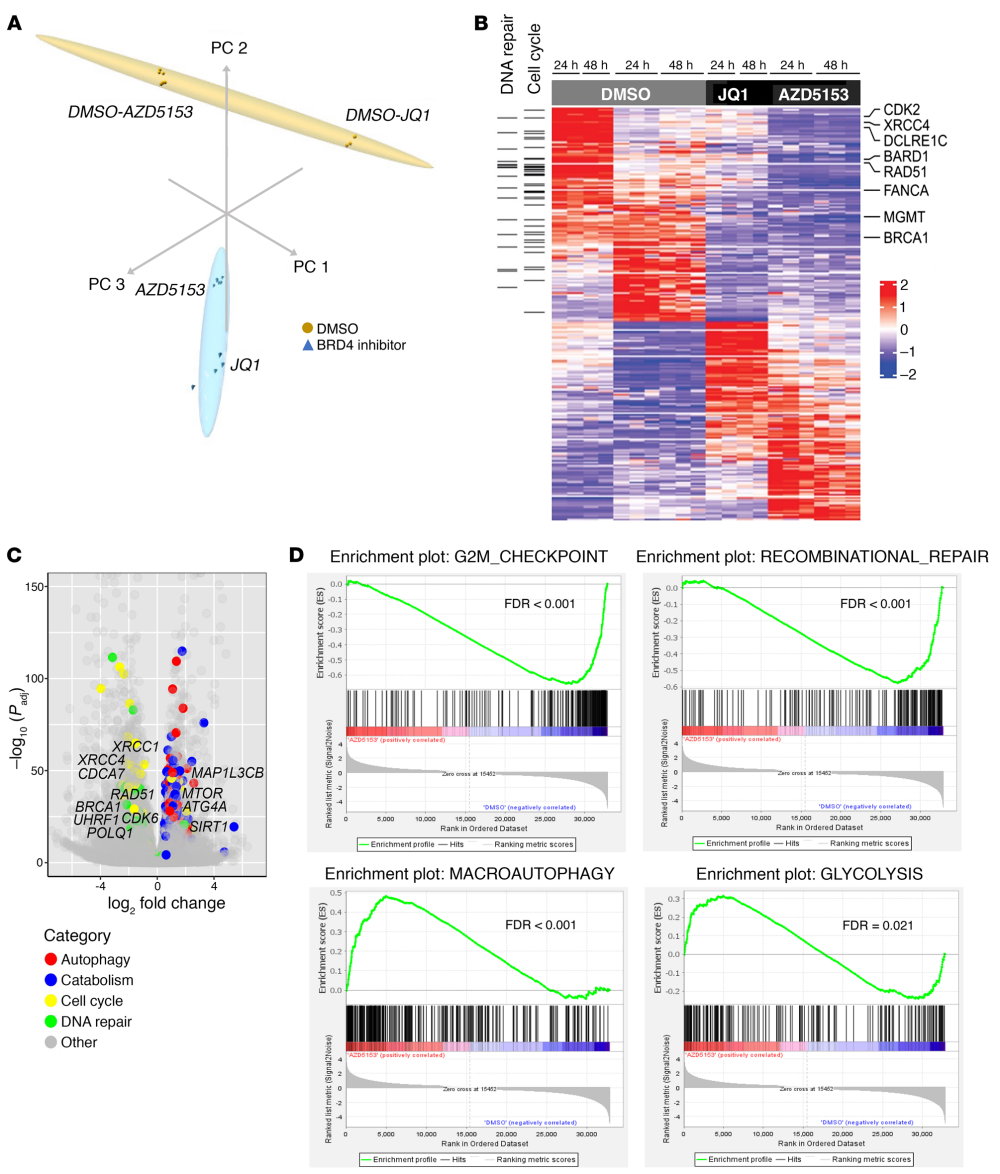
BET bromodomain inhibition altered gene expression in DMG cells. (**A**) Principal component analysis (PCA) of RNA-Seq in SF8628 DMG cells treated with 1 μM AZD5153 and 0.5% DMSO (triplicates each time point), or 300 nM JQ1 and 0.5% DMSO (duplicates each time point), for 24 and 48 hours. (**B**) Heatmap generated from RNA-Seq data, showing differentially expressed genes (*P* adj < 0.05) in SF8628 DMG cells treated with 1 μM AZD5153 and 0.5% DMSO (triplicates each time point), or 300 nM JQ1 and 0.5% DMSO (duplicates each time point), for 24 and 48 hours. Horizontal black bars to the left indicate genes involved in DNA repair and cell cycle pathways. (**C**) Volcano plot of SF8628 DMG cells treated with 1 μM AZD5153. AZD5153-treated samples are shown as dots colored according to associated pathways (*x* axis: log_2_ fold change; *y* axis: –log_10_ *P_adj_* values). (**D**) GSEA pathway analysis in AZD5153-treated SF8628 DMG cells. Significantly downregulated (FDR < 0.001, upper panels) and upregulated (macroautophagy: FDR < 0.001, glycolysis: FDR = 0.021, lower panels) pathways.

**Figure 6:**
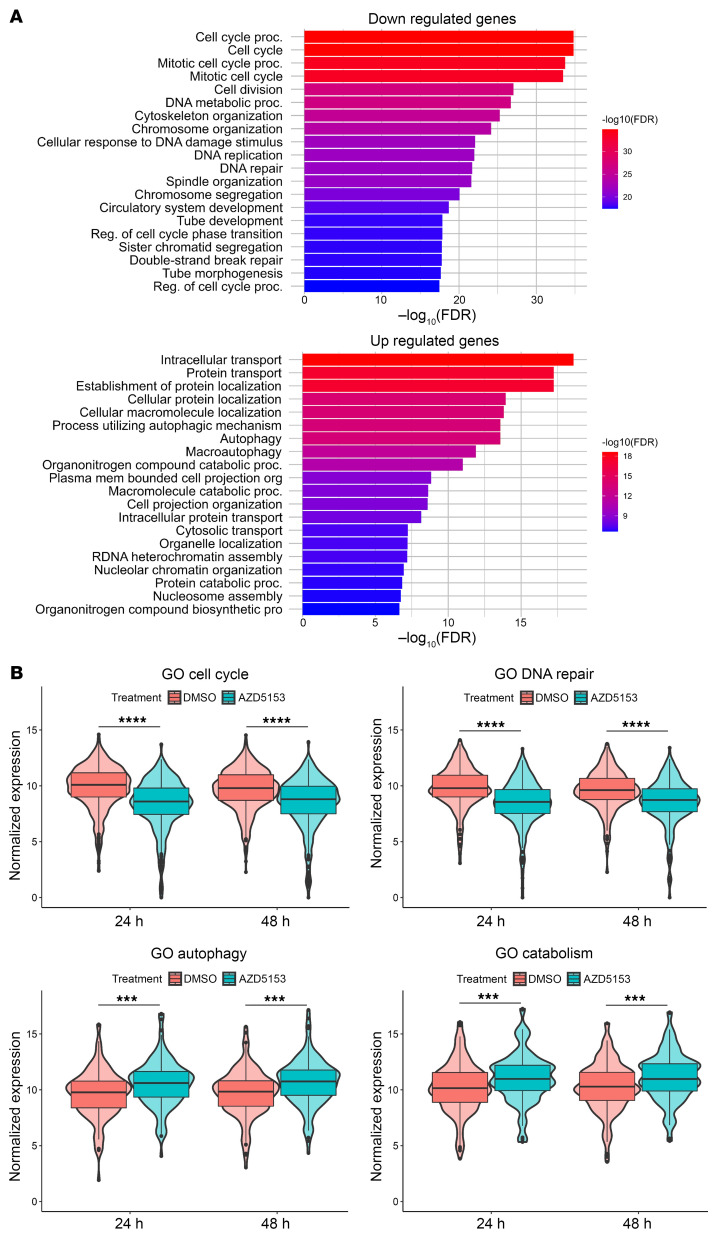
BET bromodomain inhibition altered gene sets in biological pathways in DMG cells. (**A**) GO enrichment analysis of top 20 downregulated pathways (upper panel) and upregulated pathways (lower panel) in SF8628 DMG cells treated with 1 μM AZD5153. proc., process; Reg., regulator. (**B**) Violin plots to compare the expression of the 4 gene signatures across conditions (upper left: cell cycle; upper right: DNA repair; lower left: autophagy, lower right: catabolism). Unpaired *t* test values for comparisons, each treatment, ****P < 0.0001; autophagy: ***P = 0.00025 for 24 hours, ***P = 0.00015 for 48 hours; catabolism: ***P = 0.00057 for 24 hours, ***P = 0.00032 for 48 hours.

We have previously shown that JQ1 treatment causes a change in the expression of genes that promote tumor growth in K27M-mutant DMG (15). In our current RNA-Seq analysis, we performed unsupervised principal component analysis of SF8628 DMG cells treated with DMSO and BET bromodomain inhibitors (AZD5153, JQ1) for 24 and 48 hours. We found a global gene expression shift in AZD5153-treated DMG cells compared with DMSO-treated samples (Figure 5A). We compared the RNA-Seq data from the samples treated with DMSO or AZD5153, and the previous RNA-Seq data from the samples treated with JQ1. The differentially expressed genes were highly correlated between the samples treated with JQ1 and AZD5153 (Figure 5B), including 3,301 genes upregulated and 3,591 downregulated in response to the BET bromodomain inhibitor. Gene set enrichment analysis (GSEA) (Figure 5, C and D, and Supplemental Figure 6, A and B) and gene ontology (GO) pathway analysis (Figure 6, A and B, and Supplemental Figure 6, C and D) showed that cell cycle (e.g., *CDK6*, *CDCA7*, and *UHRF1*) and DNA double-strand breaks (DSB) repair pathways (e.g., *BRCA1*, *RAD51*, *XRCC1*, *XRCC4*, and *POLQ1*) were among the most significantly downregulated with the BET bromodomain inhibitor treatment. AZD5153 and JQ1 treatments also upregulated gene pathways involved in autophagy (e.g., *ATGA4*, *MAP1LC3B*) and catabolism pathways including glycolysis and protein/macromolecule catabolic pathways (e.g., *SIRT1*, *MTOR*) (Figure 5, C and D, and Supplemental Figure 6, A and B). The senescence-associated genes *CDKN1A* and *HMGA1* were upregulated by AZD5153 treatment (Supplemental Figure 7, A and B). However, *CDKN2A* was downregulated by AZD5153 treatment. This could be due to increased H3K27me3, which repressed the PRC2 targets, including *CDKN2A* (Supplemental Figure 7, A and B).

**Figure 7:**
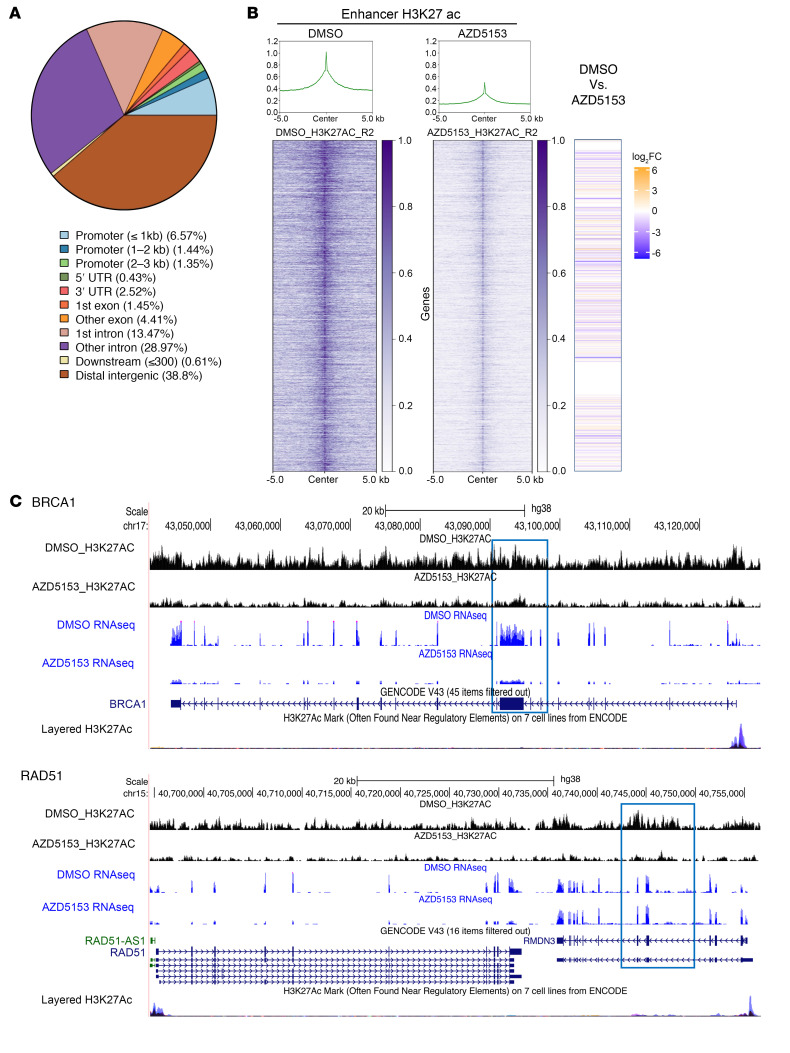
BET bromodomain inhibition altered genome-wide H3K37ac occupancy and transcription in DMG cells. CUT&RUN was performed using H3K27ac antibody in SF8628 DMG cells treated with 1 μM AZD5153 or 0.5% DMSO for 48 hours. (**A**) Pie chart showing the distribution of H3K27ac across the DMG genome. (**B**) Heatmaps showing H3K27ac occupancy with DMSO versus AZD5153 treatment. Metaplots above indicate corresponding H3K27ac occupancy. Each plot is centered on the summit of the average occupancy and extended 5 kb upstream and downstream (–5 kb and +5 kb, respectively). Corresponding gene expression at the H3K27ac binding sites generated from RNA-Seq are shown to the right. (**C**) Gene annotation tracks showing H3K27ac occupancy and gene expression for the *BRCA1* and *RAD51* loci. The enhancer region is highlighted with a square for each gene.

BET bromodomain inhibition is known to suppress gene expression by dissociating BRD from the active chromatin mark histone H3K27ac (26). We have shown that genomic occupancy of H3K27ac and BRD is required for enhancer activity and gene expression in DMG cells (15). To determine the effects of BET bromodomain inhibition on gene expression associated with H3K27ac occupancy, we performed CUT&RUN followed by next-generation sequencing in DMG cells treated with AZD5153 (Figure 7). The CUT&RUN data showed that the majority of H3K27ac enrichments occurred in introns (first intron: 13.47%; other intron: 28.97%) and intergenic regions (38.8%) (Figure 7A). Metaplots and heatmaps showed the enrichments of H3K27ac signal near the previously defined enhancer regions in DMG cells (Figure 7B). AZD5153 treatment dramatically reduced H3K27ac occupancy at enhancer regions. To investigate the enrichment of transcription factors among the H3K27ac DNA-binding sites, we used the DiffBind R package to determine differential peaks between DMSO- and AZD5153-treated samples (FDR < 0.05). We performed simple enrichment motif analysis in SF8628 DMG cells and found a significant enrichment of DNA sequencing motifs involving neuronal developmental transcriptional factors such as *LHX1–3* and *HOX13* (e-value < 0.05; Supplemental Table 3). Interestingly, the H3K27ac peaks of 2 representative DNA repair genes, *BRCA1* and *RAD51*, were diminished at enhancer regions in the SF8628 cells treated with AZD5153 (Figure 7C). Expression of these DNA repair genes was significantly downregulated in AZD5153-treated samples in the RNA-Seq analysis (Figure 5, B and C). Senescence-associated gene expression was not correlated with H3K27ac occupation (Supplemental Figure 7C). Taken together, our results suggest that BET bromodomain inhibition promotes a transcriptionally silent chromatin state by reducing H3K27ac occupancy and represses the expression of the genes involving DSB repair in K27M-mutant DMG cells.

### BET bromodomain inhibition enhances radiation-induced DNA damage.

**Figure 8:**
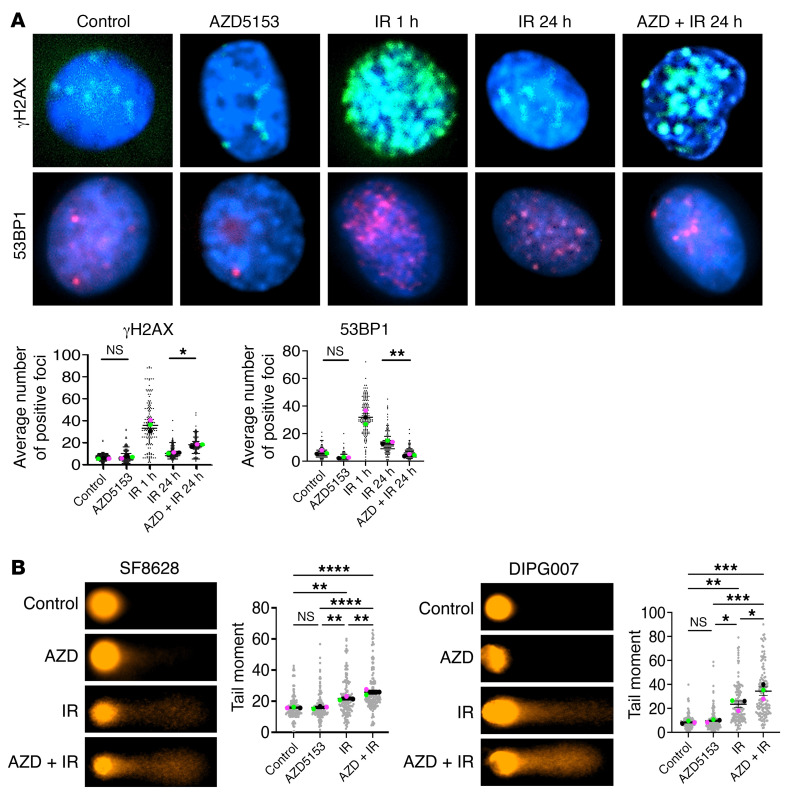
BET bromodomain inhibition enhanced radiation-induced DNA damage in DMG cells. (**A**) Effect of AZD5153 (1 μM) on formation of γH2AX and 53BP1 foci in 6 Gy–irradiated SF8628 DMG cells. Top: Representative images of nuclei from each treatment showing γH2AX and 53BP1 foci. Original magnification ×400. Bottom: Graph showing average number of γH2AX and 53BP1 foci/nucleus. Values shown are the average (mean ± SEM) from triplicate samples. One-way ANOVA comparisons between treatments: γH2AX, *P = 0.0368, IR vs. AZD5153 + IR at 24 hours; 53BP1: **P = 0.0043, IR vs. AZD5153 + IR at 24 hours. (**B**) Representative images of alkaline comet assay in SF8628 and DIPG007 DMG cells treated with AZD5153 followed by IR. Right: Bar graph showing value (mean ± SEM) from triplicate samples for each treatment for DNA damage grade sore in 50 cells. One-way ANOVA comparisons between treatments, ****P < 0.0001; SF8628: control vs. IR, **P = 0.0010; AZD5153 vs. IR, **P = 0.0012; IR vs. AZD5153 + IR, **P = 0.0095; DIPG007: control vs. IR, **P = 0.0069; control vs. AZD5153 + IR, ***P = 0.0002; AZD5153 vs. IR, *P = 0.0117; AZD5153 vs. AZD5153 + IR, ***P = 0.0003; IR vs. AZD5153 + IR, *P = 0.0398.

**Figure 9:**
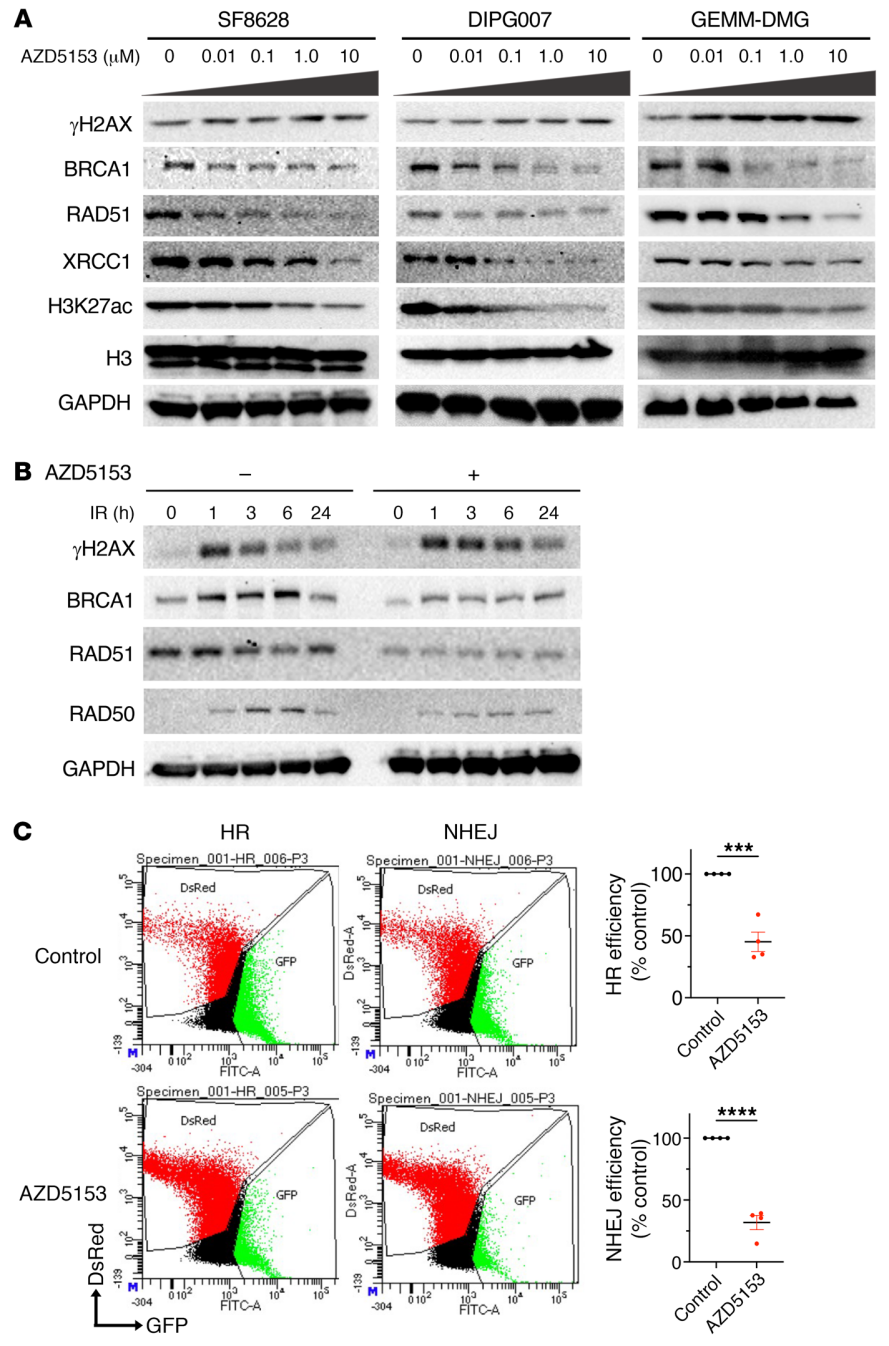
BET bromodomain inhibition induced DNA damage and suppressed DNA repair in DMG cells. (**A**) Western blotting showing the effect of AZD5153 (0–10 μM) on expression of DNA repair marker: BRCA1, RAD51, and XRCC1, DNA damage marker: γH2AX, and H3K27ac. (**B**) Western blot showing effects of AZD5153 (5 μM) on expression change over time after 6 Gy IR in SF8628 DMG cells. (**C**) DNA repair assay showing effect of AZD5153 (500 μM) on HR and NHEJ pathways in SF8628 DMG cells. Flow plots represent fluorescent signals from HR and NHEJ reporter cassettes. Repair efficiency represents the ratio of GFP^+^ to DsRed^+^ cells normalized to 100% of vehicle control (0.5% DMSO). Values (mean ± SEM) shown are based on averages from quadruplicate samples. Unpaired *t* test values for comparisons between control and AZD5153 samples: ***P = 0.0004 (HR), ****P < 0.0001 (NHEJ).

We next analyzed the effects of BET bromodomain inhibition on radiation-induced DNA damage and repair pathways in DMG cells. We examined fluorescence immunocytochemistry of the DNA DSB marker γH2AX and repair marker 53BP1 to quantify the extent of DNA damage and repair in irradiated SF8628 DMG cells in either the presence or absence of the BET bromodomain inhibitors AZD5153 (Figure 8A) and JQ1 (Supplemental Figure 4C). The number of γH2AX and 53BP1 foci increased 1 hour following IR, indicating increased DNA DSB damage and repair by IR. At 24 hours after IR, γH2AX and 53BP1 foci were largely reduced in those cells due to successful repair upon DNA damage. However, irradiated DMG cells treated with BET bromodomain inhibitors sustained high levels of γH2AX at 24 hours compared with cells treated with IR alone, while 53BPI foci were decreased (Figure 8A and Supplemental Figure 4C). Similarly, comet assays showed that IR increased comet tail formation in SF8628 and DIPG007 DMG cell lines, indicating increased unrepaired DNA damage (Figure 8B). DNA damage further increased comet tail formation in irradiated DMG cells treated with AZD5153 compared with cells treated with IR alone. These results suggest that BET bromodomain inhibition may contribute to the DNA repair process to enhance radiation-induced DNA damage. Western blotting showed that AZD5153 treatment decreased expression of the DNA repair markers BRCA1, RAD51, and XRCC1 in DMG cell lines (Figure 9A). H3K27ac was also decreased by AZD5153 in a dose-dependent manner (Figure 9A). Radiation-induced γH2X expression peaked at 1 hour following radiation (Figure 9B and Supplemental Figure 4D). Expression of BRCA1, RAD51, and RAD50 was also induced by radiation and peaked at 3–6 hours following radiation (Figure 9B). BET bromodomain inhibitors extended radiation-induced γH2X expression over 6 hours following radiation (Figure 9B and Supplemental Figure 4D). In contrast, expression of BRCA1, RAD51, and RAD50 was decreased by BET bromodomain inhibitors over the time of radiation. These results suggest that BET bromodomain inhibition extends radiation-induced DNA damage signaling by suppressing the DNA repair pathway.

DNA damage is repaired by 2 major pathways: homologous recombination (HR) and nonhomologous end-joining (NHEJ) repair (27, 28). HR repairs DNA DSB during S and G_2_ phases and provides a template for error-free repair. In contrast, NHEJ is active throughout the cell cycle and directly involves ligation of DNA ends without homology. To analyze the DNA damage repair pathways in DMG cells, we transfected GFP reconstitution reporter cassettes for HR and NHEJ (29) into SF8628 DMG cells in the presence or absence of AZD5153. AZD5153 treatment reduced DNA repair ability through both the HR and NHEJ DNA repair pathways (Figure 9C), which is consistent with the RNA-Seq results showing that AZD5153 downregulated the genes involved in both HR and NHEJ repair pathways (Figure 5). Collectively, our results indicate that BET bromodomain inhibition increased radiation-induced DNA damage by inhibiting the HR and/or NHEJ DNA repair pathway in K27M-mutated DMG cells.

### BET bromodomain inhibitors enhance radiation-mediated antitumor effects in patient-derived and genetically engineered DMG animal models.

**Figure 10:**
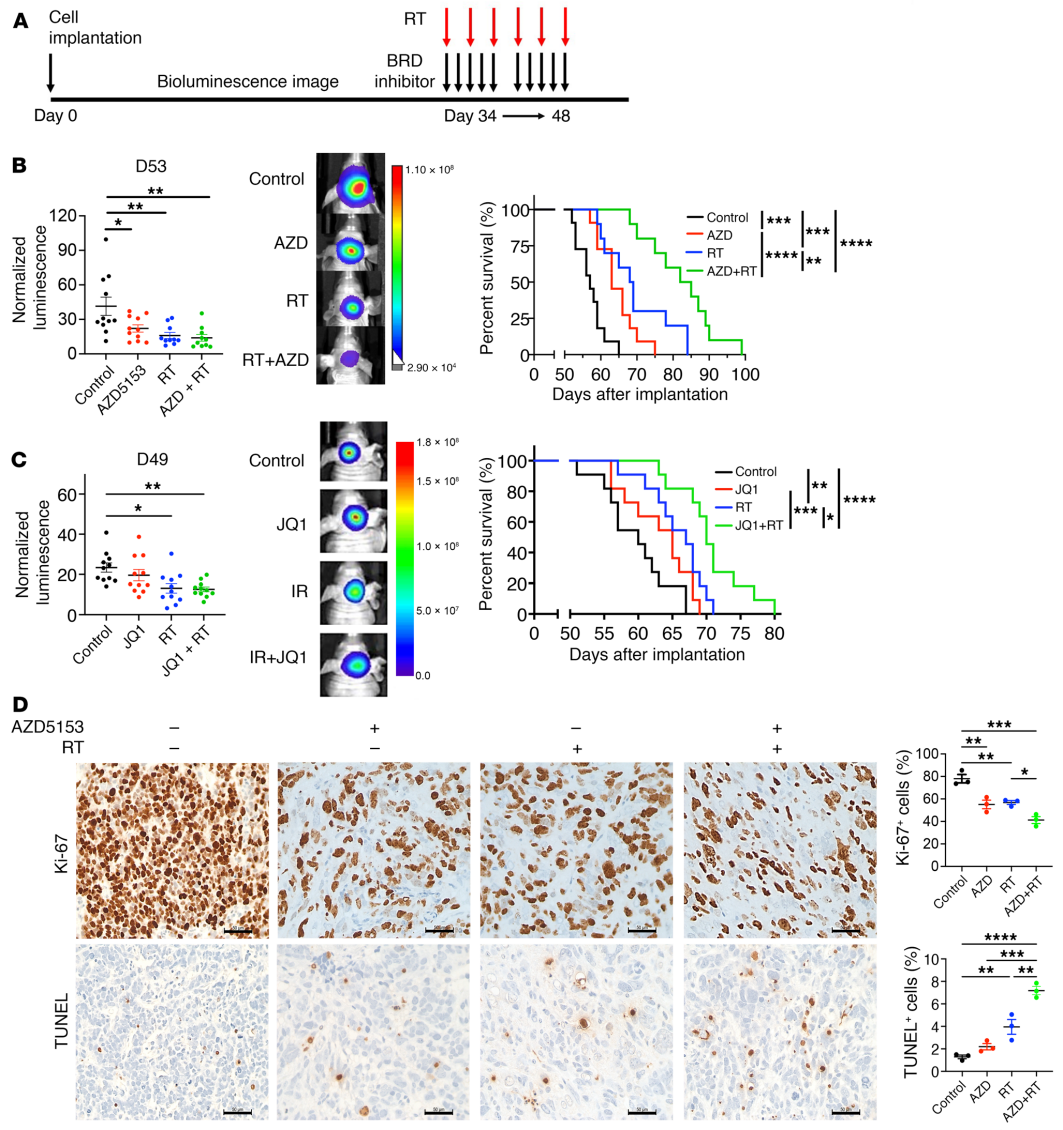
BET bromodomain inhibition enhanced radiation antitumor activity in DMG PDX models. (**A**) Experimental design for in vivo efficacy study of BET bromodomain (BRD) inhibitor in combination with radiation therapy (RT) in DMG animal models. (**B** and **C**) Mice with SF8628 PDXs were randomized to 4 treatment groups: control (DMSO, *n* = 11), AZD5153 (50 mg/kg) or JQ1 (30 mg/kg) alone (*n* = 12), RT alone (*n* = 10 for AZD study, *n* = 11 for JQ1 study), and AZD5153 + RT (*n* = 10) or JQ1 + RT (*n* = 11). Left: Dot plot representation of bioluminescence values on day 53 (AZD study) and day 49 (JQ1 study). 1-way ANOVA comparisons between treatments: AZD study, control vs. AZD5153, *P = 0.0329; control vs. RT, **P = 0.0038; control vs. AZD5153 + RT, **P = 0.0016. JQ1 study, control vs. RT, *P = 0.0128; control vs. JQ1 + RT, **P = 0.0083. Middle: Tumor bioluminescence overlay images. Right: Corresponding survival plots for each treatment. Statistical analysis using a log-rank test, ****P < 0.0001; AZD study: control vs. AZD5153, ***P = 0.0007; control vs. RT, ***P = 0.0002; RT vs. AZD5153 + RT, **P = 0.0072; JQ1 study: control vs. RT, **P = 0.0038; JQ1 vs. JQ1 + RT, ***P = 0.0006; RT vs. JQ1 + RT, *P = 0.0136. (**D**) Ki-67 and TUNEL staining for intracranial tumor at the end of treatment. Value (mean ± SEM) representing the average of positive cells in 4 high-powered fields in 3 tumor samples (*n* = 3, right). One-way ANOVA comparisons between treatments: Ki-67, control vs. AZD5153, **P = 0.0042; control vs. RT, **P = 0.0068; control vs. AZD5153 + RT, ***P = 0.0002; RT vs. AZD5153 + RT, *P = 0.0355. TUNEL, control vs. RT, **P = 0.0077; control vs. AZD5153 + RT, ****P < 0.0001; AZD5153 vs. AZD5153 + RT, ***P = 0.0001; RT vs. AZD5153 + RT, **P = 0.0024. Scale bars: 50 μm.

Based on the radiosensitizing effect of BET bromodomain inhibition on the growth of K27M-mutant DMG cells, we hypothesized that BET bromodomain inhibition increases radiation-mediated antitumor activity and survival benefit in DMG mouse models. To address this, we implanted SF8628 or GEMM-DMG cells into mouse pons and treated them with AZD5153 (50 mg/kg) or JQ1 (30 mg/kg) for 2 weeks in the presence or absence of radiation at total dose of 9 Gy (1.5 Gy per day for 3 days a week for 2 weeks) (Figure 10A). BET bromodomain inhibitor monotherapy inhibited tumor growth and extended the survival of mice with SF8628 DMG PDX (Figure 10, B and C) as well as GEMM-DMG models (Supplemental Figure 8). Similarly, radiation monotherapy provided a significant therapeutic benefit (Figure 10, B and C, and Supplemental Figure 8). We found that combination treatment with AZD5153 and radiation therapy significantly prolonged animal survival (Figure 10B and Supplemental Figure 8). Similarly, the combination treatment with JQ1 and radiation showed a significant survival benefit (Figure 10C). These in vivo efficacy studies included euthanizing the mice at the end of treatment to obtain brainstem tumor samples for analysis of tumor cell proliferation (Ki-67; Figure 10D), apoptosis (TUNEL, Figure 10D), senescence (p21, p16; Supplemental Figure 9), and migration (normal human nuclear antigen [NHNA]; Supplemental Figure 9, bottom). Analysis of intratumor Ki-67 staining showed that all therapies significantly reduced SF8628 DMG cell proliferation relative to the control group (Figure 10D). There were significantly fewer Ki-67–positive cells in the samples treated with combination therapy compared with either monotherapy. TUNEL staining results showed that the proportion of positive cells was highest in tumors derived from mice receiving combination therapy of AZD5153 and radiation relative to those receiving either monotherapy (Figure 10D). No TUNEL positivity was evident in normal brain surrounding tumor in mice receiving any of the combination treatments. Senescence marker p21 staining revealed an increase in positive cells in tumors treated with AZD5153 and in combination with radiation (Supplemental Figure 9). However, p16-positive cells were decreased by the treatment. This could be due to downregulation of the expression of the *CDKN2A* gene, which codes p16 protein. NHNA staining revealed decreasing NHNA-positive cells in the tumors of mice treated with either AZD5153 or radiation (Supplemental Figure 9). Combination treatment further decreased NHNA positive cells relative to each monotherapy.

## Discussion

**Figure 11:**
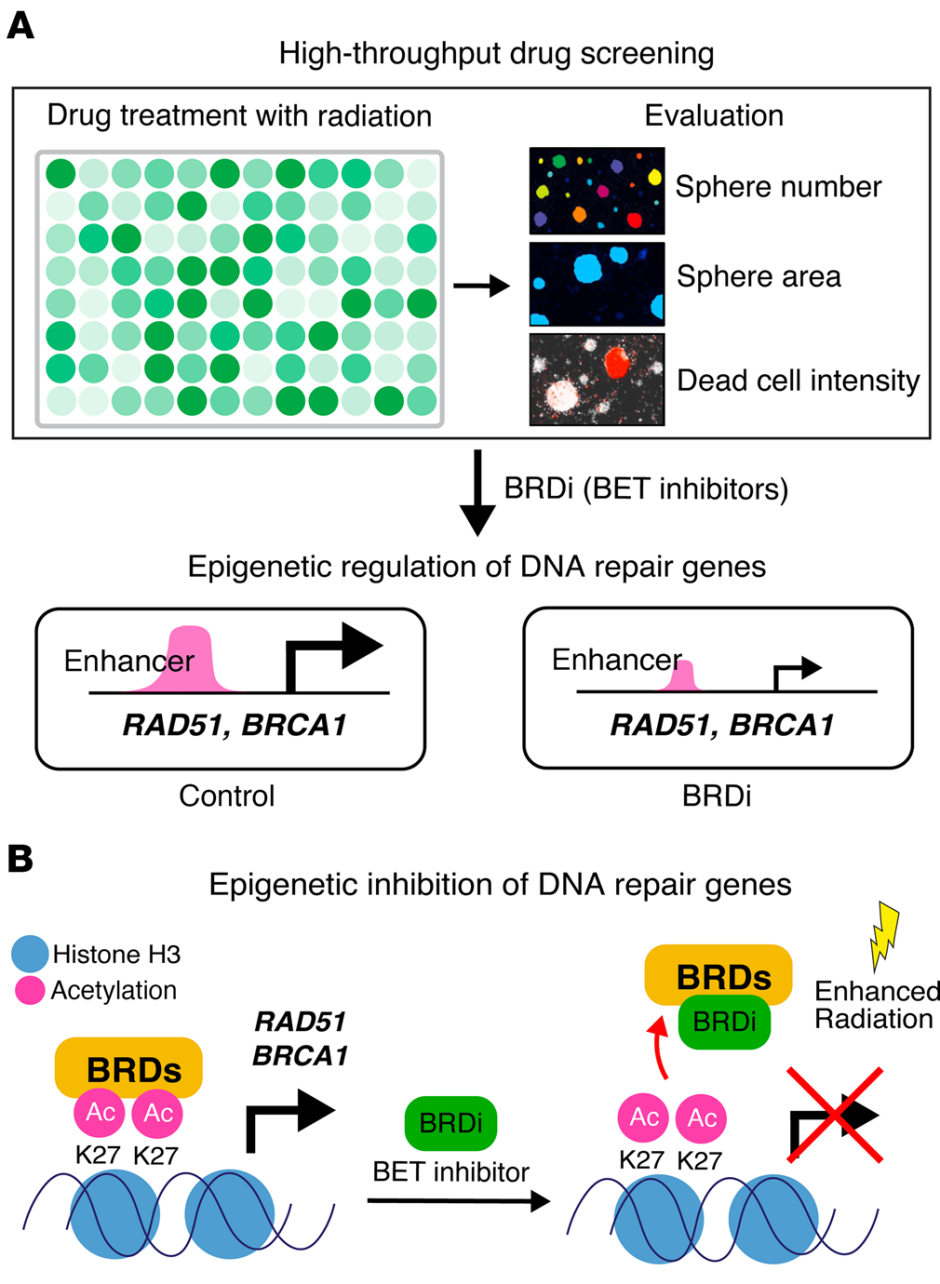
Working model. (**A**) High-throughput drug screening. BET bromodomain inhibitors (BRDi) were identified as radiosensitizers using high-throughput drug screening in the DMG cells treated with radiation. BRDi decreased H3K37ac occupancy at enhancer regions, which led to suppressed transcription involving DNA repair in DMG cells. (**B**) Epigenetic inhibition of DNA repair genes. BRDi disrupts the interaction between acetylated histone (Ac) and BRDs, inhibiting active transcription for the genes involving radiation-induced DNA damage repair, which results in enhancement of the radiation effect in DMG.

DMG is one of the most malignant childhood cancers, with a limited response to radiation therapy, resulting in a dismal prognosis: median overall survival is less than 12 months. There is a critical need for new therapeutics that enhance the effect of radiation in the treatment of DMG. Here, we identified BET bromodomain inhibitors as potent radiosensitizers in DMG using unbiased high-throughput radiosensitivity library screening (Figure 11A). High-throughput screening (HTS) is a useful tool for identifying candidate compounds from a large chemical library (30). We successfully integrated the HTS with neurosphere-based assays using automated fluorescence live-cell imaging in the presence or absence of radiation and identified several clinical-grade BET bromodomain inhibitors as top candidates for radiosensitization (Figure 1, Supplemental Figure 1, and Supplemental Table 1). In this assay, we used PI to detect the dead cell population. However, PI staining may not capture the long-term mechanism of radiation-induced cell death. Further evaluation of proliferative cell death caused by radiation, such as mitotic catastrophe (31), would be needed to understand the mechanism of radiation-induced cell death.

BET bromodomain inhibitors disrupt the binding between acetylated histone and BRD proteins and inhibit active transcription, leading to enhancement of the radiation effect in DMG (Figure 11B) (14–16). K27M-mutant DMG cells are vulnerable to BET bromodomain inhibition due to transcriptional dysregulation resulting from the mutation (14, 15, 20, 32). We demonstrated that BET bromodomain inhibition, by shRNA- or sgRNA-mediated BRD depletion (Figure 2, Supplemental Figure 2, and Supplemental Figure 3) and treatment with small molecule inhibitors (AZD5153 and JQ1) (Figure 3), suppressed the growth of human and mouse K27M-mutant DMG cells. Importantly, BET bromodomain inhibition in combination with radiation further increased radiosensitivity of DMG cells by reducing the radioresistant S-phase cell population and stemness, and increasing apoptosis and senescence (Figure 4 and Supplemental Figure 5).

DNA damage is thought to be the most important consequence of the radiation effect, and the genetic alterations of DNA repair pathways are frequently detected in pediatric high-grade glioma, including DMG (6, 10, 33, 34). We and others have demonstrated that the majority of DNA DSB caused by radiation are repaired within 24 hours of completing radiation (35–37). Thus, DNA repair is a key factor in radiosensitivity and can be a therapeutic target to enhance radiation-mediated antitumor activity in K27M-mutant DMG. Our gene expression profiling of K27M-mutant DMG cells treated with BET bromodomain inhibitors (AZD5153 and JQ1) revealed significant decreases in transcripts from the genes involved in DNA repair pathways for both HR and NHEJ, including *BRCA1*, *RAD51*, *XRCC1*, and *XRCC4* (Figures 5 and 6, and Supplemental Figure 6). To advance understanding of the transcriptional regulation in DNA repair gene pathways by BET bromodomain inhibition, we mapped genome-wide occupancy of H3K27ac in K27M-mutant DMG cells using cleavage under targets and release using nuclease (CUT&RUN; Figure 7). We have previously profiled the epigenome of K27M-mutant DMG cells and shown that K27M mutation associates with increased H3K27ac and that the heterotypic H3K27M-K27ac nucleosomes colocalize with BET BRD2 and BRD4 at the loci of actively transcribed genes (15). We analyzed the specific loci with H3K27M-K27ac occupation from the previous study (15) and found that BET bromodomain inhibition diminished a genome-wide distribution of H3K27ac at enhancer regions including 2 representative DNA repair genes, *BRCA1* and *RAD51* (Figure 7). DNA damage induces cellular senescence (38). We found that the senescence-associated genes, *CDKN1A* and *HMGA1* were upregulated by BET bromodomain inhibition (Supplemental Figure 7, A and B). However, *CDKN2A* was downregulated by BET bromodomain inhibition. There was no association between H3K27ac occupation and senescence-associated gene expression (Supplemental Figure 7C). It is possible that senescence-associated genes are controlled by different epigenetic regulation mechanisms, such as H3K27me3. Indeed, BET bromodomain inhibition reciprocally increased H3K27me3 (Supplemental Figure 2A), which resulted in silencing of PRC2 target genes such as *CDKN2A*. Our results indicated that BET bromodomain inhibitors downregulate the genes involved in DNA damage repair mediated by H3K27ac at enhancers, providing a basis for the possibility that BET bromodomain inhibitors act as radiation enhancers in DMG (Figure 7). In fact, the BET bromodomain inhibitors AZD5153 and JQ1 inhibited HR and NHEJ DNA repair pathways and prolonged radiation-induced DNA damage in K27-mutant DMG cells (Figures 8 and 9, and Supplemental Figure 4).

The molecular mechanisms of BET bromodomain inhibition in transcription and chromatin machinery for DNA damage repair in DMG are not fully understood. Upon binding to the chromatin, BET BRDs are known to function in the assembly of complexes that facilitate chromatin accessibility to transcription factors, allowing for the recruitment of RNA polymerases II (RNAPII) (39–42). In particular, BRD4 is required for subsequent progression of RNAPII through hyperacetylated nucleosomes during transcription elongation through interactions of its bromodomains with acetylated histones in order to prevent transcriptional stalling (40–42). Yaffe’s group demonstrated that deregulated transcription following inhibition or loss of BRD4 in cancer cells leads to the accumulation of RNA:DNA hybrids (R-loops) and collisions with the replication machinery causing replication stress, DNA damage, and apoptotic cell death during S phase (43). We observed that BET bromodomain inhibition decreased the radioresistant S-phase cell population, diminished self-renewal activity, and resulted in increased apoptotic cell death and cellular senescence (Figure 4 and Supplemental Figure 5). In a study of H3K27me3-deficient medulloblastoma cells (44), JQ1 inhibition sensitized medulloblastoma cells to radiation by enhancing the apoptotic response through suppression of Bcl-xL and upregulation of Bim. Loss of H3K27me3 caused an epigenetic switch from H3K27me3 to H3K27ac at specific genomic loci, altering the transcriptional profile, which associated with a radioresistant phenotype in H3K27me3-deficient medulloblastoma. Stemness is a key characteristic of radioresistance in glioma (45). BET bromodomain inhibition may sensitize H3K27me3-deficient tumors to radiation by diminishing the radioresistance phenotype and enhancing apoptotic response and cellular senescence. We will further investigate the role of BET bromodomain inhibition in the transcription machinery associated with histone modification of H3K27me3 and H3K27ac to understand the radiation-induced DNA damage response in DMG.

Consistent with in vitro experiments, our animal studies demonstrated that the combination therapy with BET bromodomain inhibitor and radiation inhibited tumor growth and increased survival in human and murine DMG mouse models compared with either therapy alone (Figure 10). The improvement resulting from the combination therapy proved to be modest. One limitation to the in vivo efficacy of BET bromodomain inhibitors is poor brain penetration (Supplemental Table 2). To increase drug concentrations in the brain, we would further investigate new drug delivery systems such as disrupting the BBB using focused ultrasound (46, 47) or bypassing the BBB using convection-enhanced delivery (48) and intranasal delivery (49). Nevertheless, our findings support the possible use of BET bromodomain inhibitor to increase the radiation-mediated antitumor effect for the treatment of DMG.

## Methods

Further information can be found in Supplemental Methods.

### Sex as a biological variable.

Our study examined 6-week-old female athymic mice (rnu/rnu genotype, BALB/c background). The animals were purchased from Envigo and housed under aseptic conditions. The animals are well established and were used to develop DMG PDXs in our published studies (15, 35, 37, 50, 51). There are no reported sex differences among DMG patients.

### Xenograft studies.

Mice received 1 μL SF8628 or GEMM-DMG cell suspension (100,000 cells/μL) by injection into the pontine tegmentum at a depth of 5 mm from the inner base of the skull, as previously described (15, 35, 37, 51, 52). For the efficacy study of AZD5153 and radiation, animals were randomized into 4 treatment groups: (i) vehicle control (0.5% hydroxy methylcellulose, 0.1% Tween 80 for AZD5153, 1% DMSO for JQ1; *n* = 11); (ii) ADZ5153 (oral gavage of 50 mg/kg; *n* = 11) or JQ1 (i.p. injection of 30 mg/kg; *n* = 11) treatment 5 times a week for 2 consecutive weeks; (iii) radiation monotherapy (1.5 Gy 3 times a week for 2 consecutive weeks, for a total dose of 9 Gy; *n* = 10 for AZD5153 study, *n* = 11 for JQ1 study); and (iv) combination therapy with AZD5153 or JQ1 and radiation (*n* = 10 for combination with AZD, *n* = 11 for combination with JQ1). Biweekly bioluminescence imaging was used to monitor tumor growth and response to therapy, as previously described (15, 35–37, 50, 51). Mice were monitored daily and euthanized at end points, which included irreversible neurological deficit or body condition score less than 2.

### Cell sources and propagation.

The SF8628 (H3.3K27M DMG) cell line was obtained from the University of California San Francisco (UCSF) Medical Center and in accordance with an institutionally approved protocol. Establishment of SF8628 cell culture from surgical specimens as well as tumor cell modification for expression of firefly luciferase for in vivo bioluminescence imaging have been described (15, 35, 37, 50, 51). DIPG007 (H3.3K27M DMG) cell line was provided by Angel Montero Carcaboso (Hospital Sant Joan de Déu, Barcelona, Spain). SU-DIPG4 (H3.1K27M DMG) and SU-DIPG36 (H3.1K27M DMG) cell lines were provided by M. Monje (Stanford University, Stanford, California). NHAs and human astrocytes expressing WT (Astro-WT) and the K27M *H3F3A* transgene (Astro-KM) have been previously described (8, 51). The SF8628 and human astrocyte cells were propagated as monolayers in complete medium consisting of DMEM (catalog 11965092) supplemented with 10% FBS (A31604–02) and nonessential amino acids (catalog 11140–050) from ThermoFisher Scientific. DIPG007, SU-DIPG4, SU-DIPG36 cell lines were grown in tumor stem medium (TSM) as neurosphere culture or with 5% FBS as adherent culture. TSM base was prepared using the following: Neurobasal-A medium (catalog 10888–022), DMEM/F-12 medium (catalog 11330–032), HEPES buffer (catalog 15630–080), sodium pyruvate (catalog 11360–070), MEM nonessential amino acids (catalog 11140–050), GlutaMAX-I supplement (catalog 35050–061), antibiotic-antimycotic (catalog 15240–096), and B-27 supplement minus vitamin A (catalog 12587–010) from ThermoFisher Scientific; EGF and FGF (catalog 100–26 and 100–146) and PDGF-A and PDGF-B (catalog 100–16 and 100–18) from Shenandoah Biotechnology; and 0.2% heparin (catalog 07980) from STEMCELL Technologies. H3.3K27M-mutant neurosphere cells were derived from a genetically engineered mouse model of DMG (GEMM-DMG) (Ntv-a; p53fl/fl; PDGFB; H3.3K27M; Cre) (48). GEMM cells were cultured under neurosphere conditions in DMEM supplemented with 10% proliferation supplement (STEMCELL Technologies), 1% Pen–Strep (Invitrogen), 20 ng/mL human basic FGF (Invitrogen), 10 ng/mL human EGF, and 2 μg/mL heparin. We used the PowerPlex 16 HS System (Promega DC2101) to analyze short tandem repeats (STRs) in order to confirm the identity of the cell lines. All cells were cultured in an incubator at 37°C in a humidified atmosphere containing 95% O_2_ and 5% CO_2_ and were mycoplasma-free at the time of testing with a Mycoplasma Detection Kit (InvivoGen).

### shRNAs and sgRNA treatments.

BRD4 and scrambled control shRNAs (BRD4 shRNAs: V3THS_378004, V3THS_326487, V3THS_326484, Control shRNA: RHS4346; Dharmacon IDs) were used to generate lentivirus and infected tumor cells according to the manufacturer’s instructions. At 24 hours after lentiviral infection, cells were selected using 2 mg/mL puromycin for 5 days prior to in vitro assays. We also generated sgRNAs to knock out BRD2, BRD3, and BRD4 expression. sgRNA for the ROSA26 gene was used as control (see Supporting Data Values). The lentiCRISPRv2 vector (a gift from F. Zhang; Addgene plasmid 52961) was digested with BsmBI and inserted the sgRNAs into the vector (53). The ligation reactions were transfected into Stbl3 cells. Positive clones were confirmed with Sanger sequence. These plasmids were cotransfected into HEK293T cells with psPAX2, pMD2.G with PEI reagents (23966, Polysciences). Supernatants containing virus particles were collected at 48 and 72 hours and were used to infect DMG cells. After 48 hours of lentiviral infection, cells were selected with 2 μg/mL puromycin for 5 days prior to the in vitro assay.

### Clonogenic survival assay.

Six-well tissue culture plates were seeded with 400–10,000 cells and allowed to adhere for 12 hours. The modified cells with BRD4 shRNAs or sgRNAs, or unmodified cells treated with 50 nM AZD5153 or 50–100 nM JQ1 alone, were irradiated at doses of 0.5, 1, 2, 3, 4, 6, and 8 Gy. Radiation was delivered by a gamma irradiator. Cells were incubated at 37°C for 2 weeks, after which colonies were counted following staining with 0.05% crystal violet. Plating efficiencies were calculated as the ratio of the number of colonies formed to the number of cells seeded. Colonies of more than 50 cells were used to indicate surviving fractions. Surviving fractions were calculated as the plating efficiency of treated cells divided by the plating efficiency of control cells. DEFs were calculated as the ratio of the dose with radiation alone to the dose with radiation and BRD4 inhibition at 10% survival.

### DNA repair assays.

GFP reconstitution reporter cassettes for detection of HR and NHEJ have been previously reported (29, 34). Plasmids containing HR or NHEJ reporter cassettes were linearized and transfected into cells to measure HR or NHEJ as a function of GFP expression. Transfections were performed using Lipofectamine 2000 (ThermoFisher Scientific 11668027). Cells with integrated reporter constructs were selected by adding 1 mg/mL geneticin (ThermoFisher Scientific 10131–035). HR or NHEJ cassette–expressing cells were treated with 1 μM AZD5153 for 72 hours, then transfected with a mixture of 5 μg ISceI-expressing plasmid and 2 μg pDsRed2-N1 (Clonetech 632406). Four days following transfection, cells were harvested, suspended in PBS, and placed on ice. Cells were then analyzed by FACS using LSRFortessa cell analyzer (BD Biosciences). Cells expressing GFP, pDsRed2-N1, or no fluorescent protein were used as calibration controls. Data were analyzed using FlowJo software. DNA repair efficiency was determined as a ratio of GFP^+^ to DsRed^+^ cells normalized to 100% of vehicle control (DMSO).

### Comet assay.

Cells were treated with 500 nM AZD5153 or 0.5% DMSO, followed by 4 Gy irradiation, and alkaline comet assays were performed (54). Brieﬂy, 10,000 cells were resuspended in 75 μL of 0.5% (wt/vol) low-melting-point agarose and pipetted on slides precoated with 1.5% (wt/vol) normal-melting-point agarose. Coverslips were placed on top to spread the cell suspension evenly, and the slides were incubated on ice for 10 minutes. Next, the slides were slowly immersed into ice-cold, freshly made lysis buffer for 1 hour. After removal from the lysis buffer, the slides were placed into an electrophoresis tank filled with alkaline buffer (4°C) for 20 minutes, and electrophoresis was performed for 20 minutes at 300 mA. Slides were then removed, and drops of neutralization buffer were added 3 times. Finally, the slides were stained with PI (20 μg/mL). Images were observed under a ﬂuorescence microscope. The DNA damage in more than 50 cells for each experimental condition was quantified by determining the tail moment, a function of both the tail length and the intensity of the DNA in the tail relative to the total DNA, by using OpenComet score software (55).

### RNA-Seq and analysis.

Cells were cultured as described above and treated with vehicle or 1 μM AZD5153 for 24 and 48 hours. RNA was extracted using the RNeasy Mini Kit (QIAGEN) according to the manufacturer’s instructions. Paired-end (150 bp) FASTQ files for the AZD5153-treated and DMSO-treated samples were obtained from Novogene, and read quality and absence of adapter sequences were verified using FastQC. Single-end (50 bp) FASTQ files for JQ1-treated and DMSO-treated samples were obtained from the NCBI Gene Expression Omnibus database (GEO GSE78801, SRA SRP071040). FASTQ files were aligned to the hg38 genome using RNA-STAR (56), and aligned reads were counted using HTSeq-count (57). HTSeq-count files were imported into R (https://www.r-project.org/), and differential expression analysis was performed with the DESeq2 package (58) using default settings. For the combined JQ1 and AZD5153 analysis, the samples were normalized to stably expressed genes to reduce batch effect (ENSG00000085978, ENSG00000103275, ENSG00000110442, ENSG00000157764, ENSG00000169951, ENSG00000170832, ENSG00000235859). DESeq2 normalized reads were imported into GSEA v4.2.3 (https://www.gsea-msigdb.org/gsea/index.jsp) (59), and GSEA was run using MSigDB v2023.1 with the following parameters: permutations = 1,000, permutation type = gene set, enrichment statistic = weighted, gene ranking metric = signal2noise, max size = 500, min size = 15, normalization mode = meandiv. Gene ontology (GO) analysis was performed using ShinyGO 0.76.2 (60). Principal component analysis was conducted in R using the pca3d package, and heatmaps were generated using the ComplexHeatmap R package (61). Enriched genes from GO or GSEA were used to generate violin plots. Violin and volcano plots were generated in R using ggplot2.

### CUT&RUN assay.

CUT&RUN was performed as previously described (62, 63). Cells were treated with 500 nM AZD5153 or 0.5% DMSO for 48 hours, and 500,000 cells were harvested and fixed with freshly prepared 0.5% PFA for 2 minutes. After cross-linking was stopped using 500 μL of 2.5 M glycine, cells were washed 3 times with digitonin wash buffer (20 mmol/L HEPES, pH 7.4, 150 mmol/L NaCl, 0.5 mmol/L spermidine, protein inhibitor cocktail, 0.02% digitonin, 0.05% SDS, and 1% Triton X-100) and bound to Concavalin A–coated beads prewashed with binding buffer. Cells on beads were then incubated with antibodies on a nutator overnight at 4°C (1:500 for H3K27ac antibody; Cell Signaling Technology 8173) After overnight incubation, cells were washed 3 times with 0.02% digitonin wash buffer, resuspended in 100 μL digitonin buffer containing protein A–micrococcal nuclease fusion protein (pA-MNase) and antibody complex assemblies (pA-MNase and antibodies were preassembled at a 2:1 ratio in 50% glycerol at 4°C for 1 hour) and nutated at 4°C for 1 hour. After pA-MNase–second antibody binding, cells were washed 3 times with digitonin wash buffer for 5 minutes. Finally, cells were resuspended in 100 μL ice-cold digitonin buffer in a heating block precooled in the ice water bath. Digestion of the chromatin was initiated by the addition of 2 mmol/L CaCl_2_ in the tube and lasted for 60 minutes at 0°C. The digestion was stopped by addition of 100 μL 2× STOP buffer (340 mmol/L NaCl, 20 mmol/L EDTA, 4 mmol/L EGTA, 0.02% digitonin, 100 μg/mL RNase A, and 50 μg/mL glycogen). Digested DNA was released by incubation at 37°C for 30 minutes, and the supernatant was collected. Then 5 μL proteinase K and 200 μL 2× elution buffer (20 mmol/L Tris-HCl [pH 8.0], 300 mmol/L NaCl, 20 mmol/L EDTA, 2% SDS, 10 mmol/L DTT) was added to each sample for reverse cross-linking at 65°C overnight. DNA was extracted by phenol-chloroform and dissolved in 12 μL low-EDTA TE buffer. Libraries were prepared by the ACCEL-NGS 1S plus DNA library kit, and samples were sequenced using an Illumina NextSeq 500 platform. Paired-end (100 bp) FASTQ files were obtained from MedGenome. Adapter and quality trimming, alignment, normalization (CPM), and peak calling (SEACR) were performed using the nf-core/cutandrun pipeline (64). Consensus peaks were imported into R, and differential analysis was conducted using DiffBind v3.6.5. Peak annotation and generation of pie charts to visualize genomic peak locations were performed using ChIPseeker v1.22.1 (65). The peaks shown in Figure 6B were identified from a previous publication (15). FASTQ files for 2 H3K27M ChIP-Seq samples and corresponding control samples were obtained from NCBI GEO series GSE78801 and were processed using the nf-core/chipseq pipeline with default parameters. Consensus peaks were filtered to include the top 5,000 enriched regions. These regions were used to generate heatmaps and metaplots for AZD5153- and DMSO-treated samples using computeMatrix (referencePoint option), plotHeatmap, and plotProfile from deeptools v3.5.1 (66).

### Statistics.

Survival plots were generated and analyzed using the Kaplan-Meier method and Graph-Pad Prism v9.5 software. Differences between survival plots were estimated using a log-rank test with Holm’s adjustment. For other analyses, 1-way ANOVA was applied for multiple-group comparison with a post hoc Tukey’s test and a 2-tailed unpaired *t* test for comparison in 2 groups using the Prism software.

### Study approval.

All animal protocols were approved by the Northwestern University Institutional Animal Care and Use Committee.

### Data availability.

All data are available from the corresponding author and are provided in the Supplemental Supporting Data Values file. RNA-Seq and CUT&RUN sequencing data were deposited and available in the NCBI’s Gene Expression Omnibus database (GEO GSE236598).

## Author contributions

JW, MRC, NT, MN, ZZ, OJB, and RH designed the study. JW performed the majority of the experiments, and JW and RH wrote the manuscript. JW, TS, EU, KA, YI, OJB, and RH performed and analyzed the in vivo experiments. JW and YM performed CUT&RUN. MRC and MJG performed and analyzed the high-throughput drug screening. SG performed all bioinformatics analyses and provided interpretation of the data. JW, TS, EU, SK, and NT performed and analyzed the apoptosis assay and interpreted the data. JW, TS, EU, and YI performed and analyzed the immunohistochemistry studies. OJB provided clinical supervision in the interpretation of data. ZZ, MN, OJB, and RH provided supervision in the interpretation of data. All authors commented on the manuscript and approved the included data.

## Supplementary Material

Supplemental data

Unedited blot and gel images

Supporting data values

## Version 1 05/21/2024

In-Press Preview

## Version 2 07/01/2024

Electronic publication
