# Supplementary material for: BET bromodomain inhibition potentiates radiosensitivity in models of H3K27-altered diffuse midline glioma
Source: J Clin Invest. 2024 May 21;134(13):e174794. doi: 10.1172/JCI174794 (PMC11213469; doi:10.1172/JCI174794)
Supplement: Supplemental data [file jci-134-174794-s205.pdf]

SUPPLEMENTAL METHODS

High-throughput drug screening

Cells were expanded as neurospheres, digested to a single cell suspension using Accutase (Stem Cell), and plated at 10,000 cells per well in 384-well imaging plates (Greiner Bio-One). After 4 days in culture, the neurospehere cells were treated with 5 μM final concentration of compounds or 0.05% DMSO-matched control from a small molecule screening library containing 1,280 FDA-approved drugs and 1,600 clinical-stage compounds (MedChemExpress). After 6-hour incubation, replicate plates were irradiated with 10 Gy (Rad Source RS-2000) or mock irradiated. Treated neurospheres were cultured for another 72 hours, and subsequently stained with propidium iodide (2 μg/ml, Thermo Scientific) and Hoeschst 33342 (6 μg/ml, Thermo Scientific) for at least 2 hours at 37 °C. Plates were then imaged on an ImageXpress Micro Confocal high content imaging system (Molecular Devices). Neurospheres in each well were identified using a minimum threshold for nuclear staining intensity and size. Within each neurosphere mask, the average pixel intensity of the propidium iodide channel was computed and averaged across all neurospheres from the well to obtain a measure of neurosphere cell death. For each compound, cell death was normalized to the DMSO-treated control wells. The values of no irradiation (0 Gy) versus 10 Gy irradiation were compared to identify radiosensitizers.

Cell Viability Assays

For determination of cell viability effects of BET bromodomain inhibition, tumor cells were seeded in 96-well plates, at 3000 cells per well, and cultured in the presence of 0-10 μM of AZD5153 and JQ1 for 72 hours with triplicate or quadruplicate samples for each incubation condition. Relative numbers of viable cells were determined using CellTiter 96 AQueous One Solution Cell Proliferation Assay (Promega). The 50% growth inhibition (IC_{50}) values were calculated using nonlinear least-squares curve-fitting. The proliferation effects by BET bromodomain inhibition were analyzed on days 1, 2, 3, 4, and 5 in the presence of vehicle (0.5% DMSO) and IC_{50} concentrations of AZD5153 and JQ1, or BRD4 shRNAs and sgRNAs for 4-5 days.

Colony Formation Assays

Colony formation assays were performed by plating at 2,500 cells/ml in 60-mm plates or a 6-cm dish. The cells were allowed to adhere for 24 hours and then treated with 0.5% DMSO or IC_{50} values of AZD5153. Cells were incubated at 37°C for 2 weeks and colonies were counted following staining with 0.05% crystal violet. The modified cells with BRD4 shRNAs and sgRNAs were also seeded at 2,500 cells/ml in 60-mm plates and incubated for 2 weeks for colony formation.

Flow Cytometry

The effects of BET bromodomain inhibition on cell proliferation were determined by BrdU incorporation into the DMG cells modified with BRD4 sgRNAs or treated with 500 nM of AZD5153 in combination with radiation (4-6 Gy). The cells were plated into 6-cm dishes and allowed to adhere overnight. Samples were then treated with AZD5153 only, radiation only, or combination of AZD5153 with radiation. Inhibitor treatments were initiated concurrently with radiation. Unmodified cells or vehicle (0.5% DMSO) treatment were used as a control. After 48 hours, cells were fixed in 80% ethanol and stained with 7-amino-actinomycin D (7-ADD). Cells were then subjected to flow cytometric analysis using a BD FACSCalibur instrument and data were analyzed using FlowJo 8.8 software. To measure cell size, consecutive side scatter area (SSC-A) over forward scatter (FSC)-area, and FSC-height over FSC-area gates were used to isolate single cells. Then, G1 cells were gated by DNA content with 7-ADD. Finally, we compared cell size using the gating based on mean SSC-A signal.

Apoptosis Assay

For analysis of apoptosis, the BD Pharmingen FITC Annexin V Apoptosis Detection Kit I (BD Biosciences, San Jose, CA) was used according to the manufacturer's instructions. Briefly, cells were seeded into 6-well plates. The following day, cells were treated with 0.5% DMSO or 1 μM of AZD5153 and/or radiation (4-6 Gy). Radiation was delivered by gamma irradiator. After 48 hours, cells were

harvested using trypsin, washed three times with phosphate-buffered saline (PBS), and stained with propidium iodide (PI) staining solution and FITC Annexin V. The stained samples were analyzed using flow cytometry (BD LSRFortessa Analyzer cytometer) and the percentage of apoptotic cells (Annexin V-positive and Annexin V + PI double-positive) was quantified using FlowJo software (version 10.5).

Immunoblotting

Cells were cultured and treated with shRNAs, sgRNAs, AZD5153, or radiation as described above. Total cell lysate was collected from the cell pellets at each condition using lysis buffer (ThermoFisher 89900) supplemented with 1% protease (ThermoFisher 78430) and 1% phosphatase (ThermoFisher 78420) inhibitor cocktails, resolved by sodium dodecyl sulfate polyacrylamide gel electrophoresis, and then immunoblotted using standard techniques. Transfer was conducted using 1x NU-Page transfer buffer (ThermoFisher NP0006–1) with 10% methanol. The protein samples were transferred to Immuno-Blot PVDF membranes (Bio-Rad 1620177) for 90 minutes at 40V. The membranes were blocked for 1 hour at room temperature in 5% skim milk in Tris-HCl buffered saline with 0.05% Tween 20 (TBST), and then probed with primary antibodies (1:1000 dilution) for overnight at 4°C, washed three times in TBST, incubated with horseradish peroxidase-conjugated secondary antibody (1:1000 dilution), followed by washing in TBST and then in phosphate-buffered saline (PBS), and signals visualized by enhanced chemiluminescence (ECL) (ThermoFisher 32106). The primary antibodies used were BRD2 (D89B4, #5848), BRD4 (E2A7X. #13440), phospho-BRCA1(pBRCA1, Ser1524, #9009), phospho-Rad50 (pRad50, Ser635, #14223), Rad51 (D4B10, #8875), phospho-Histone H2A.X (γH2AX, Ser139, 20E3, #9718), p21 (12D1, #2947), GAPDH (14C10, #2118), acetyl-histone H3 (H3K27ac, D5E4, #8173), histone H3 (1B1B2, #14269) and secondary antibody (Anti-Rabbit IgG, #7074P2) (Anti-Mouse IgG, #7076P2) were obtained from Cell Signaling Technologies (Danvers, MA). BRD3 (2099C3a, ab50818), XRCC1 (EPR4389, ab134056) was obtained from Abcam. BRCA1 (BRCA1, 07-434) was obtained from Sigma.

Immunocytochemistry

50,000 cells were seeded into 8 well chamber slides and incubated with AZD5153 for 24 hours and then irradiated with 6 Gy. 4% paraformaldehyde (PFA, Polysciences 18814) was used to fix tumor cells on cover slips at 1 and 24 hours after radiation. Slips were then rinsed in PBS and blocked with 0.3% Triton X-100 and 5% normal goat serum (Cell Signaling 5425) in PBS for 1 hour at room temperature. Coverslips were incubated overnight at 4°C with γH2AX (Ser139, 20E3, Cell Signaling 9718) or 53BP1 (Cell Signaling 4937) antibody at 1:250 dilution in PBS containing 0.3% Triton X-100 and 5% normal goat serum followed by 50-minute incubation at room temperature in anti-rabbit antibody Alexa Fluor 488 (Cell Signaling 4412) or anti-rabbit antibody Alexa Fluor 555 (Cell Signaling 4413) at 1:1000 dilution in PBS with 5% normal goat serum for single reporter labeling. The coverslips were rinsed in PBS four times and nuclei were stained by incubating the coverslips at room temperature in the dark with 4',6-diamidino-2-phenylindole (DAPI, Cell Signaling, 8961) diluted at 1:500 in PBS, followed by additional rinses in PBS and sterile water. The cover slips were then mounted on glass slides using Vectashield (Vector Laboratories H-1200) and analyzed with a Carl Zeiss Axioimager 2 microscope (Zeiss, Oberkochen, Germany). Foci were counted in 100 cells per treatment condition.

Beta galactosidase assay

Cellular senescence was measured using a β-Galactosidase (β-gal) staining assay kit (Cell Signaling) following manufacturer's instruction. 10,000 cells were seeded in a 6-well plate and allowed to proliferate. After 24 hours seeding, cells were treated with 50nM of AZD5153 or 0.5% DMSO followed by 4Gy ionizing radiation (IR). After 2 days culture with fresh medium, cells were fixed and stained with β-gal reagent at pH 6.0, overnight at 37°C. Development of a blue color indicative of senescing cells was visualized and quantified under a microscope.

Sphere formation assay

All cell cultures were used with neurospheres culture conditions for this assay. Single-cell suspensions from 3D neurospheres of each cell culture were plated into U-shaped non-attachment coating

96-well plates (Thermo Scientific, #174925) with various seeding densities (0, 1, 2, 5, 10, and 50 cells per well). After 24 hours seeding, cells were treated with 50nM of AZD5153 or 0.5% DMSO followed by 4Gy IR. The plates were incubated at 37 °C for 10 days. On day 10, each well was observed under a 10 × magnification for the determination of individual tumor cell spheres formation (> 40 µm). "Positive" wells (containing spheres) were counted, log fraction (vs wells w/o spheres) was plotted, and data were analyzed using the extreme Limiting Dilution Analysis (http://bioinf.wehi.edu.au/software/elda/index.html). For measuring sphere size, 100 cells were seeded in each well, and the size was measured 10 days after treatment with IC_{50} of AZD5153 with and without IR.

Quantitative PCR

10,000 cells were seeded with six-well tissue culture plates. The cells were allowed to adhere for 24 hours and then treated with 500nM of AZD5153 with and without IR. After 24 hours, cells were collected, and mRNA was isolated using RNeasy Mini Kit (QIAGEN). DNA was generated using SuperScript IV (Invitrogen) and 20 ng was used for qRT-PCR amplification using maxima SYBR green master mix (Invitrogen). Gene expressions were normalized with that of *GAPDH* endogenous control gene. The relative level of each gene was calculated using the $2^{-\Delta\Delta Ct}$ method and reported as relative fold-change mean ± S.E.M. The primer sequence of *GAPDH*, *CDKN2A*, and *CDKN1A* genes were described in Supporting Data values.

Radiation therapy

Radiation therapy (RT) has been used to treat intracranial PDX mouse model as previously described (35-37). Briefly, Mice were anesthetized with an intraperitoneal (IP) injection of a mixture containing ketamine and xylazine and placed in the radiation chamber. Radiation was derived by a Gammacell 40 Exactor (Best Theratronics, LtD). Mice were shielded with lead blocks to limit radiation to mice brainstem and reduce the dose to the mice's eye and body. The treatment regimen of radiation was described in Methods.

Immunohistochemistry (IHC)

Brains were collected from the mice at 3 hours following completion of the last treatment (n=2 for each treatment). Paraformaldehyde-fixed brains were paraffin-embedded and sectioned (10 μm) for hematoxylin and eosin (HE) and anti-Ki67 (1/100, ab15580, Abcam). To assay apoptotic response to treatment, TUNEL staining was performed using the DeadEnd Colorimetric TUNEL system (Promega) according to the manufacturer's protocol. To evaluate senescence and migration of tumor cells, the brain sections were stained with primary antibodies of p21 (1/100, NBP2-48351, Novus), p16 (1/100, ab211542, Abcam), and normal human nuclear antigen (NHA) (1/100, MAB4383, Sigma), respectively. All images were taken at 40x magnification.

Analysis of drug concentrations in the brain

Athymic mice (n = 3) were administered the test drugs for 3 consecutive days and euthanized 1 hour after the third administration. The brainstem was dissected from the surrounding brain, the serum was collected by cardiac puncture, and the samples were snap frozen and stored at -80°C. The test drugs were extracted from homogenized tissues using a Bullet Blende (Next Advance, Troy, NY, USA). Homogenates were extracted with organic solvent and transferred to an autosampler for liquid chromatography-mass spectrometry (LC/MS) analysis (VP Series 10 System; Shimadzu, Kyoto, Japan) for determination of the drug content (Integrated Analytical Solutions, Berkeley, CA, USA). The brain penetration ratio was calculated as the value of the drug concentration in the brain divided by the serum concentration.

Supplemental Table 1.

Detected Compound	Status	Developer	Fold Δ#
I-BET762	Phase 1/2	GSK	10.8
BMS-986158	Phase 1	BMS	9.5
AZD5153	Phase 1	AstraZeneca	6.9
GS-5829	Phase1	Gilead	7.9
ABBV-075	Phase1	AbbVie	7.8

Supplemental Table 1. Top clinical BET bromodomain inhibitors identified as radiosensitizers in high-throughput drug screening. Fold Δ#: [Effect of drug+10 Gy – Effect of drug]/[Effect of DMSO+10 Gy – Effect of DMSO]

Supplemental Table 2.

Drug concentration (mg/ml)	JQ1	AZD5153	BMS-986158
Brainstem	2.40 ± 1.53	3.06 ± 1.14	0.63 ± 0.20
Serum	4.83 ± 2.89	23.4 ± 6.57	10.85 ± 3.15
**Brain penetration ratio (%)**			
Brainstem / Serum	47.7 ± 7.9	12.9 ± 1.25	5.02 ± 1.32

Supplemental Table 2. Drug concentration in brainstem and serum.

Supplemental Table 3.

Motif	Logo	3 Top hits in databases
**oligos_6nt_mkv4_m1oligos_6nt_mkv4_m1**	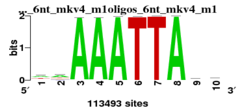	**<u>versus Homer:</u> homer_150_Lhx1, homer_152_Lhx3, homer_151_Lhx2,**
**oligos_6nt_mkv4_m2oligos_6nt_mkv4_m2**	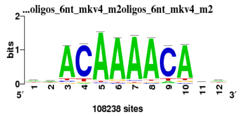	**<u>versus Homer:</u> homer_130_HOXB13, homer_133_HOXD13,**
**oligos_6nt_mkv4_m3oligos_6nt_mkv4_m3**	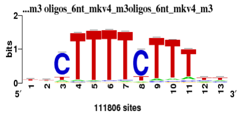	**<u>versus Homer:</u> homer_280_T1ISRE,**
**oligos_6nt_mkv4_m4oligos_6nt_mkv4_m4**	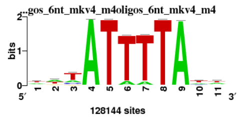	**<u>versus Homer:</u> homer_30_Cdx2, homer_31_CDX4, homer_130_HOXB13,**
**oligos_6nt_mkv4_m5oligos_6nt_mkv4_m5**	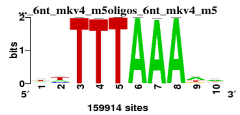	**<u>versus Homer:</u> homer_36_CHR, homer_153_Unknown, homer_125_Hnf1,**
**positions_6nt_m1positions_6nt_m1**	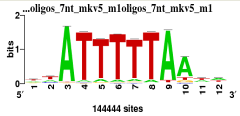	**<u>versus Homer:</u> homer_5_AGTAAACAAAAAAGAACANA,**
**positions_6nt_m2positions_6nt_m2**	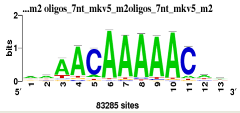	**<u>versus Homer:</u> homer_99_Fra2, homer_12_Atf3, homer_141_GATGACTCATCN,**
**positions_6nt_m3positions_6nt_m3**	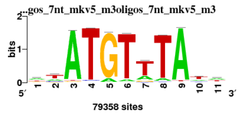	**<u>versus Homer:</u> homer_163_Mef2c, homer_164_Mef2d, homer_161_Mef2a,**
**positions_6nt_m4positions_6nt_m4**	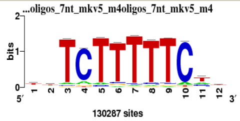	**<u>versus Homer:</u> homer_161_Mef2a, homer_162_Mef2b, homer_163_Mef2c,**
**positions_6nt_m5positions_6nt_m5**	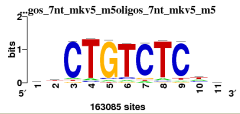	**<u>versus Homer:</u> homer_47_DMIR1, homer_48_DMIR6, homer_139_Isl1,**

Supplemental Table 3. The motifs enriched among H3K27ac DNA binding sites down-regulating by AZD5153 treatment in K27M-mutant DMG cells.

Supplemental Figure 1.

A

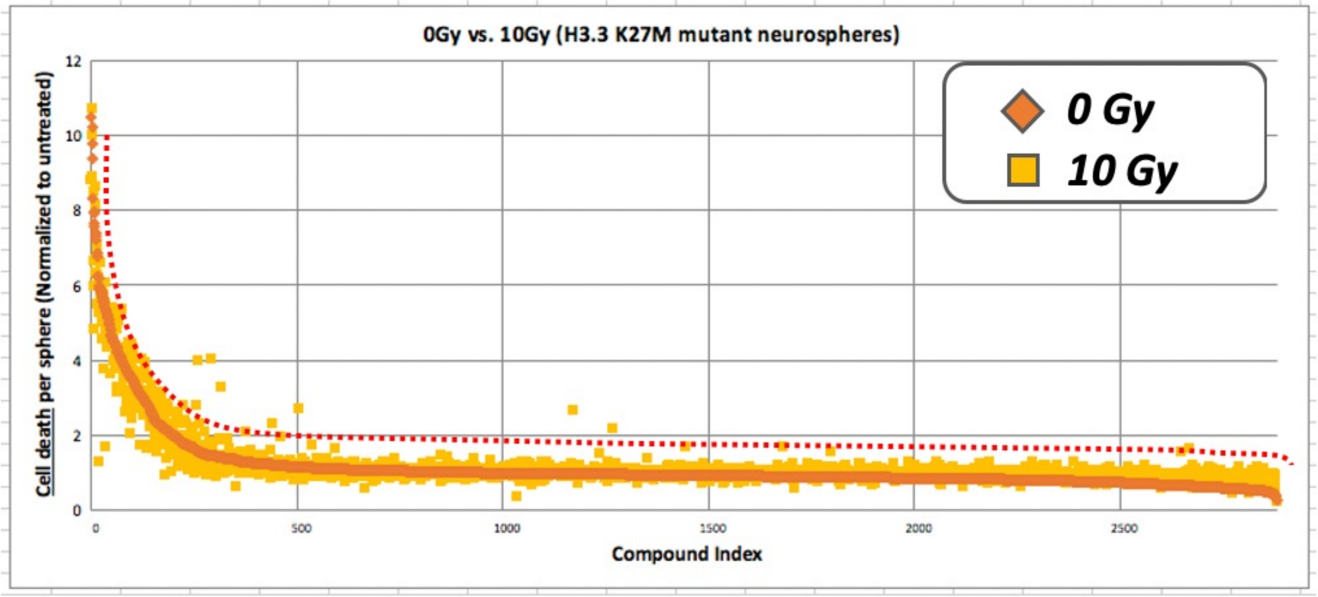

B

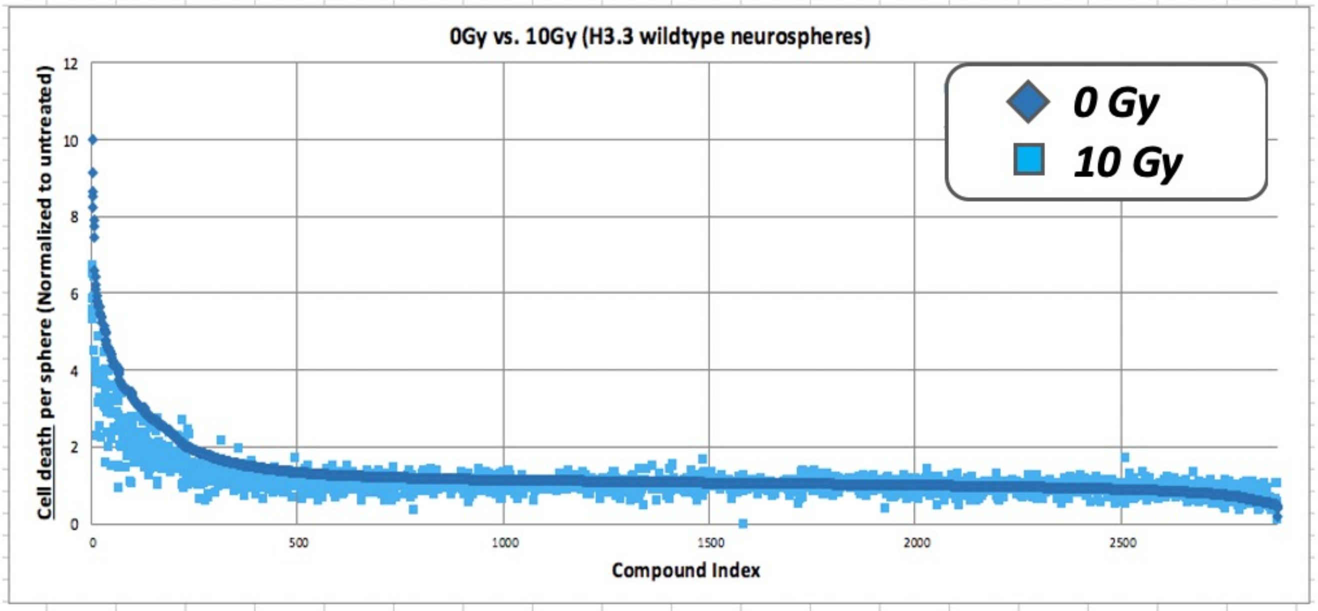

Supplemental Figure 1. High-throughput drug screening with radiation identified BET bromodomain inhibitors as radiosensitizers in H3.3 K27M-mutant and wild type (WT) DMG cells. Tumor cells isolated from GEMM-H3.3 K27M DMG (Ntv-a; p53fl/fl; PDGFB; H3.3K27M; Cre) (**a**) and GEMM-H3.3 WT DMG (Ntv-a; p53fl/fl; PDGFB; H3.3WT; Cre) (**b**) were cultured ex vivo as neurospheres and used to drug screening for radiosensitizers. The drugs above the red dot line are candidates of radiosensitizer.

Supplemental Figure 2.

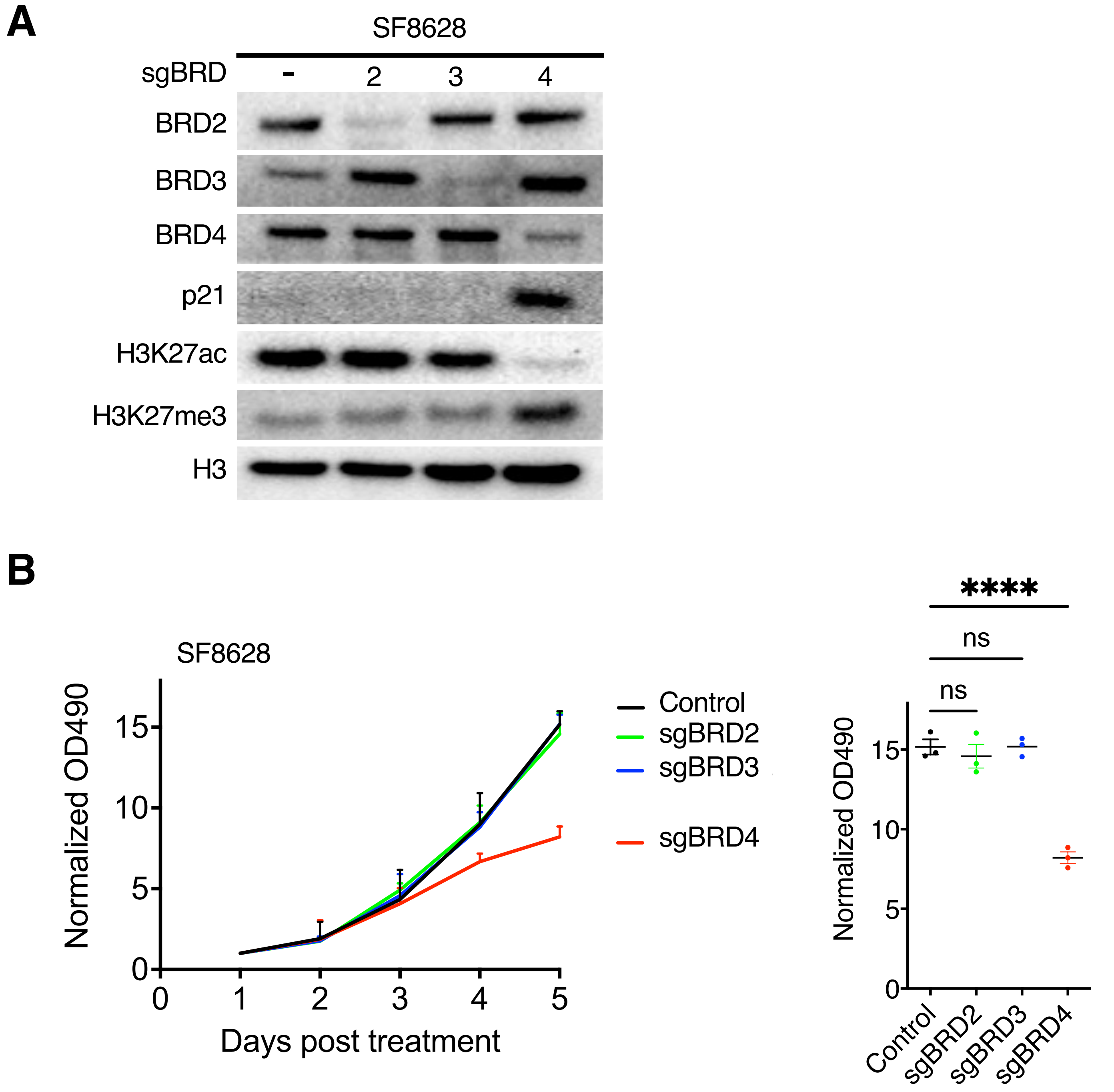

Supplemental Figure 2. Effects of BRDs depletion on DMG cell. (**a**) Western blot results showing effects of sgRNA-mediated depletion of BRD proteins in SF8628 cells. (**b**) Cell growth plot showing anti-proliferative effects of sgBRD2, sgBRD3, and sgBRD4 in SF8628 cells based on the absorbance values (OD 490) measured each day. Values shown are the average (mean ± SEM) from triplicate samples for each condition as day 1 normalized. Dot plot representation of normalized OD 490 values on day 5 (right). One-way ANOVA comparison test was performed using compared to Control.

Supplemental Figure 3.

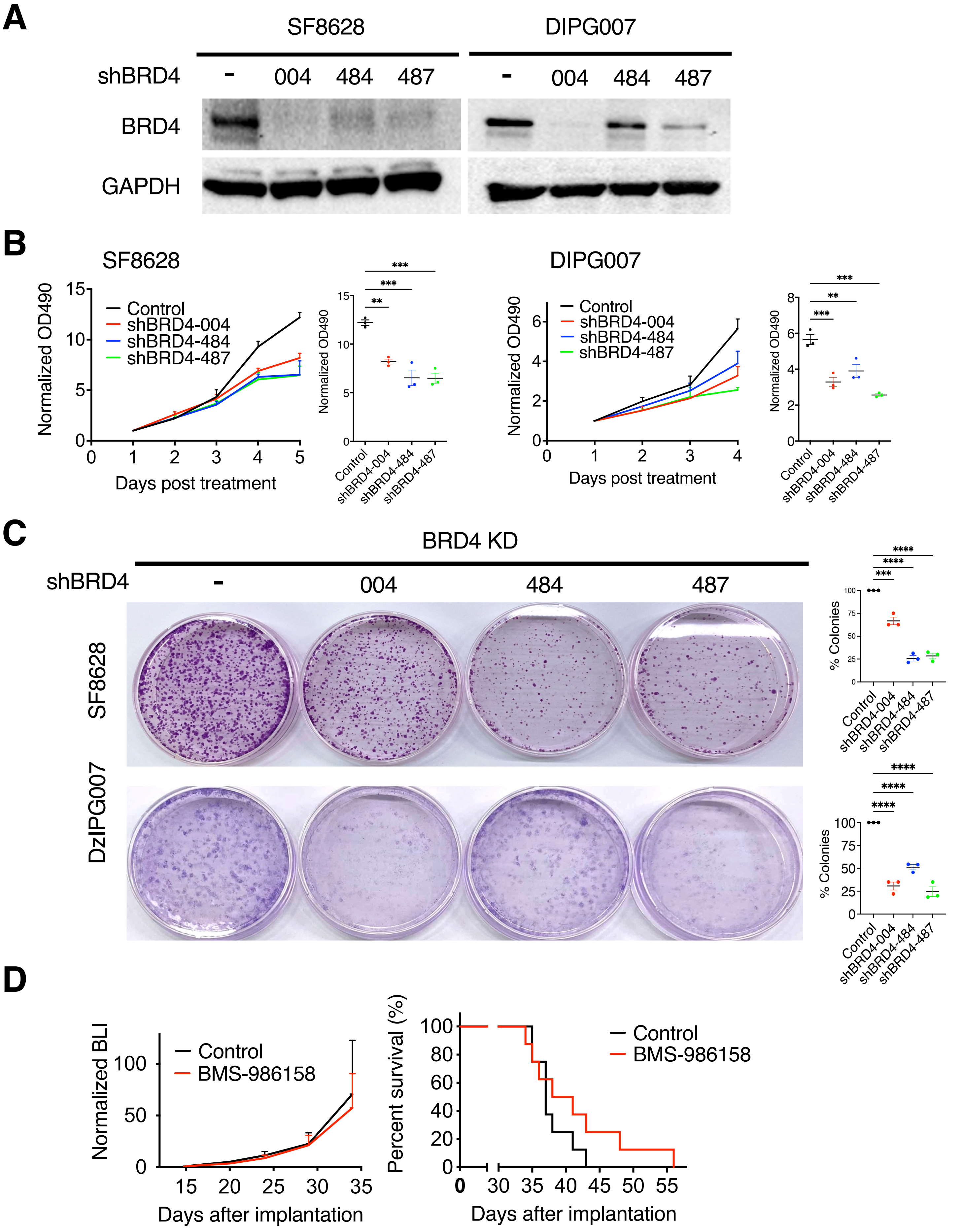

Supplemental Figure 3. Knockdown effects of BRD4 shRNA on DMG cell proliferation and in vivo efficacy of BMS-986158 BET bromodomain inhibitor in DMG PDX model. (**a**) Western blot results showing effects of shRNA-mediated depletion of BRD4 (shBRD4) in SF8628 and DIPG007 cells. (**b**) Cell growth plot showing anti-proliferative effects of shBRD4 in SF8628 and DIPG007 cells based on the absorbance values (OD 490) measured each day. Values shown are the average (mean ± SEM) from triplicate samples for each condition as day 1 normalized. Dot plot representation of OD 490 values on day 5 (right). Statistical analysis was performed using One-way ANOVA comparison test compared to Control: SF8628, shBRD4-004 ***P* = 0.0014; shBRD4-484 ****P* = 0.0001; shBRD4-487 ****P* = 0.001; DIPG007, shBRD4-004 ****P* = 0.0006; shBRD4-484 ***P* = 0.0040; shBRD4-487 ****P* = 0.0001. (**c**) Effects of shBRD4 on colony formation in SF8628 and DIPG007 cells. Bar graph representation of colony numbers in DMG cells (right). Values shown are the average (mean ± SEM) from triplicate samples for each condition. One-way ANOVA comparisons between the control (scramble shRNA) and shBRD4: **** indicates *P* < 0.0001; SF8628, shBRD4-004 ****P* = 0.0002. (**d**) Mice with SF8628 intracranial tumors were either treated with vehicle (DMSO, n = 8) or BMS-986158 (25 mg/kg for 5 days a week for 2 consecutive weeks, n = 8). Left: Growth curve showing tumor bioluminescence image (BLI) of the mice for each treatment group as normalized by BLI on day 15 when treatment was initiated. Values shown are the average (mean ± SD) from each mouse. Right: Corresponding survival plots of each treatment group.

Supplemental Figure 4.

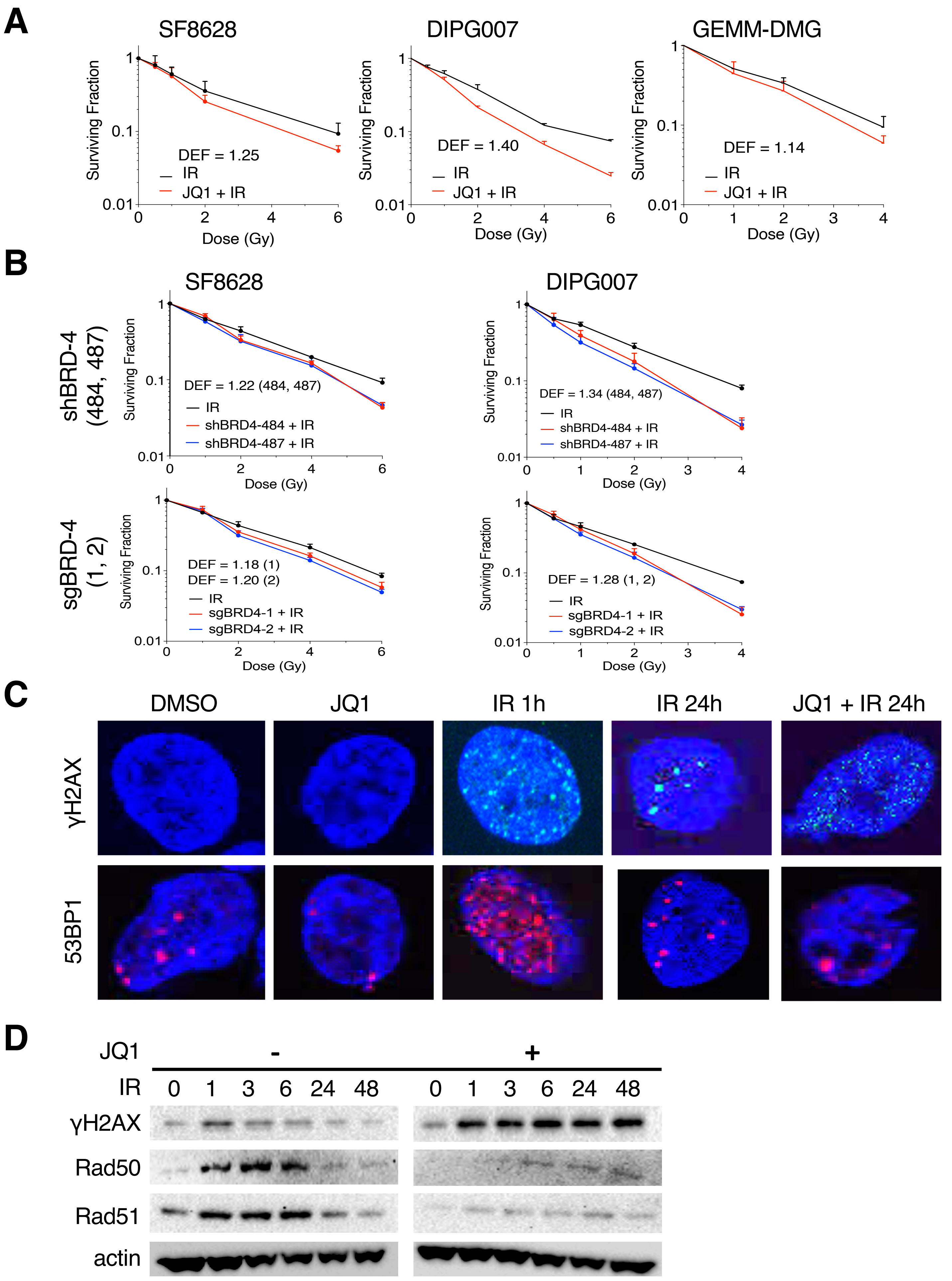

Supplemental Figure 4. Effects of BET bromodomain inhibition on radioresponse and radiation-induced DNA damage in DMG cell. Clonogenic survival results for (**a**) K27M-mutant DMG cells (SF8628, DIPG007, GEMM-DMG) treated with JQ1 (100 nM for SF8628 and DIPG007, 50 nM for GEMM-DMG) and (**b**) K27M-mutant DMG cells (SF8628, DIPG007) modified with shBRD4 (upper) and sgBRD4 (lower) prior to ionizing radiation (IR). Survival fractions, shown as (mean ± SEM) based on averages from triplicate samples, were normalized to plating efficiency for each cell lines. Dose Enhancement Factor (DEF) for each cell line was calculated at 10% survival level. (**c**) Effect of JQ1 (0.5 μM) on γH2AX and 53BP1 foci formation in 6 Gy irradiated SF8628 DMG cells. Representative images of nuclei from each treatment, showing γH2AX (upper) and 53BP1 (lower) foci. (**d**) Western blot results showing effects of JQ1 at 0.5 μM on expression change of DNA damage marker: γH2AX, DNA repair marker: RAD50 and RAD51 over time after 6 Gy IR in SF8628 DMG cells.

Supplemental Figure 5.

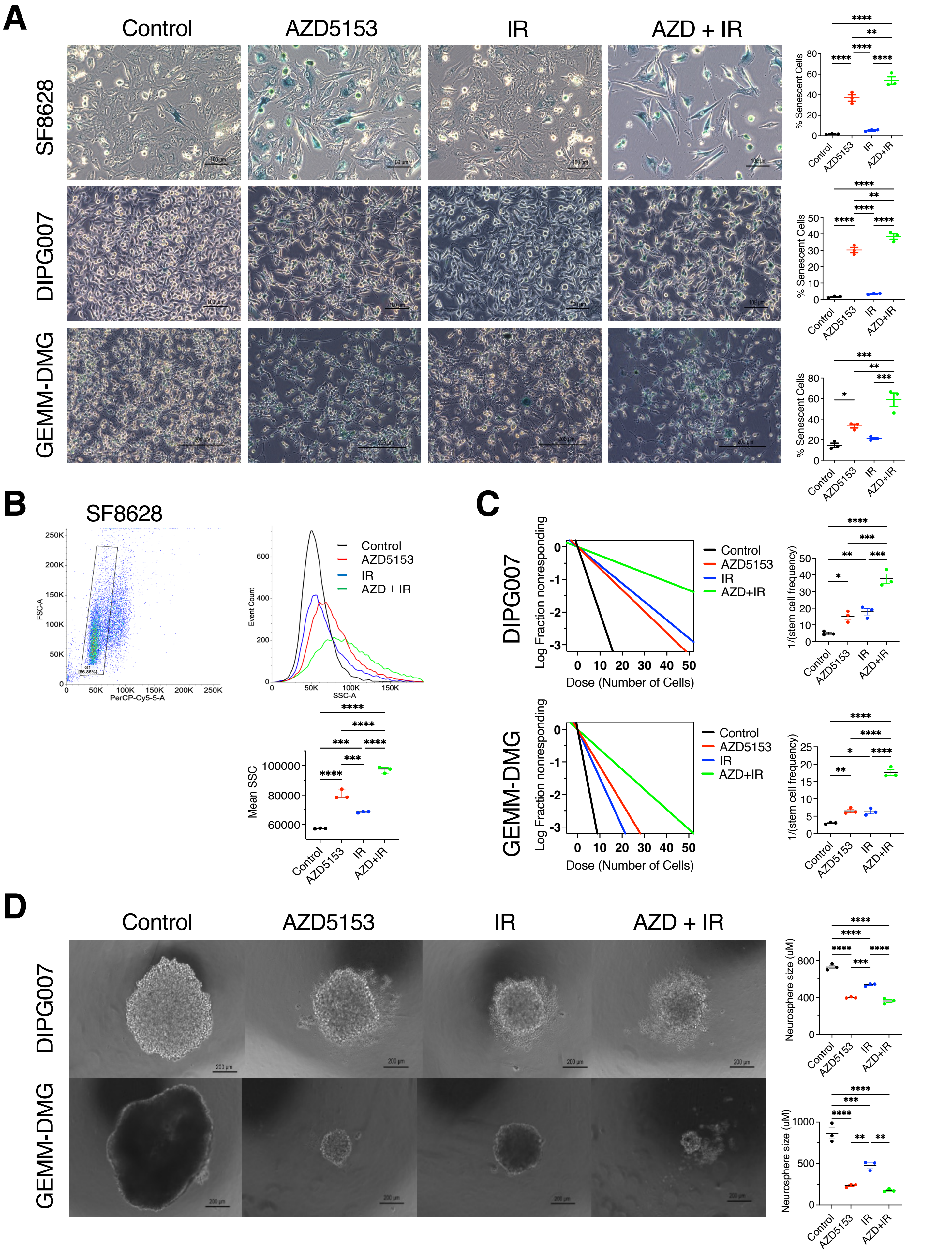

Supplemental Figure 5. Effects of BET bromodomain inhibition on senescence and stemness in DMG cell. (**a**) Senescence-associated beta-galactosidase staining (blue color) was evaluated after 50 nM AZD5153 or 0.5% DMSO (control) treatment followed by 4Gy IR. The percentage of beta-galactosidase positive cells was quantified in the different experimental groups by cell counting. Magnification used was 400×(10×40). Values shown are the average (mean ± SEM) from triplicate samples for each condition. One-way ANOVA comparison test values between each treatment: **** indicates $P < 0.0001$; AZD5153 vs. AZD5153 + IR, $^{**}P = 0.0056$ (SF8628); AZD5153 vs. AZD5153 + IR, $^{**}P = 0.0057$ (DIPG007); control vs. AZD5153, $^{*}P = 0.0260$ (GEMM-DMG); AZD5153 vs. AZD5153 + IR, $^{**}P = 0.0045$ (GEMM-DMG). (**b**) Cell size was measured by flow cytometry. SF8628 cells were gated by G1 DNA content and sorted into three size bins based on the side scatter parameter (SSC) as a proxy for cell size. The mean value of SSCA was quantified in the different experimental groups. Values shown are based on averages from triplicate samples, and error bars represent SEM (mean ± SEM). One-way ANOVA comparison test value between each treatment: **** indicates $P < 0.0001$; control vs. IR, $^{***}P = 0.0005$; AZD5153 vs. IR, $^{***}P = 0.0003$ (SF8628). (**c**) Stem-like cell self-renewal activity was evaluated by extreme limiting dilution analysis (ELDA). DMG cells were grown in neurosphere culture and treated with 50 nM AZD5153 or 0.5% DMSO (control) treatment followed by 4Gy IR. On day 10, neurosphere cells were counted, and log fraction was plotted. Dot plot representation of the reciprocal of stem cell frequency calculated on day 10 (right). Values shown are the average (mean ± SEM) from triplicate samples for each condition. One-way ANOVA with comparison test values between each treatment: **** indicates $P < 0.0001$; DIPG007, control vs. AZD5153, $^{*}P = 0.0270$; control vs. IR, $^{**}P = 0.0077$; AZD5153 vs. AZD5153 + IR, $^{***}P = 0.0002$; IR vs. AZD5153 + IR, $^{***}P = 0.0005$; GEMM-DMG, control vs. AZD5153, $^{**}P = 0.0099$; control vs. IR, $^{*}P = 0.0153$. (**d**) Representative images of DMG neurosphere cells (DIPG007 and GEMM-DMG) with and without 50 nM AZD5153 or 0.5% DMSO (control) treatment followed by 4Gy IR after 10 days. Magnification used was 400×(10×40). Values shown are based on diameter average (mean ± SEM) from triplicate samples. One-way ANOVA comparison test values between each treatment: **** indicates $P < 0.0001$; DIPG007, AZD5153 vs. IR, $^{***}P = 0.0003$; GEMM-DMG, control vs. IR, $^{***}P = 0.0004$; AZD5153 vs. IR, $^{**}P = 0.0075$; IR vs. AZD5153 + IR, $^{**}P = 0.0020$.

Supplemental Figure 6.

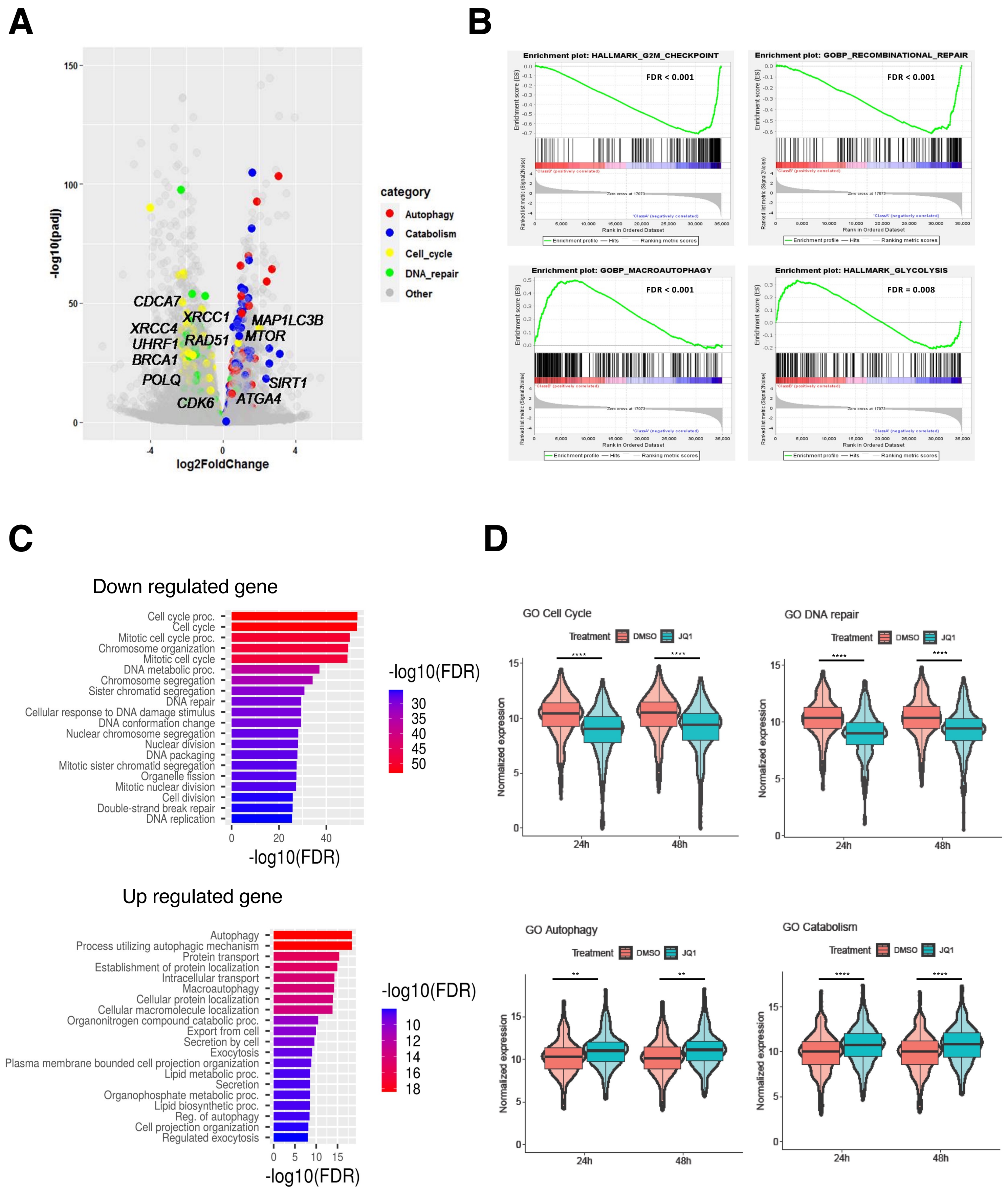

Supplemental Figure 6. Effects of JQ1 BET bromodomain inhibitor on gene expression in DMG cell. (**a**) Volcano plot of SF8628 DMG cells treated with 0.3 μM JQ1 or 0.5% DMSO. DMSO-treated samples and JQ1 treated samples are shown as colored dots and colored by associated pathways (x-axis: $\log_2$ Fold Change; y-axis: $-\log_{10}$ padj values). (**b)** GSEA pathway analysis in JQ1 treated SF8628 DMG cells. Significantly down-regulated (FDR < 0.001, upper panel) and up-regulated (Macroautophagy: FDR < 0.001, Glycolysis: FDR = 0.008, lower panels) pathways. (**c)** GO enrichment analysis of top 20 down-(negative) regulated pathways (upper panel) and up-(positive) regulated pathways (lower panel) in SF8628 DMG cells treated with 0.5 μM JQ1 or 0.5% DMSO. (**d**) Violin plots to compare the expression of the four gene signatures across conditions (upper left: cell cycle; upper right: DNA repair; lower left: autophagy, lower right: catabolism). Unpaired t-test values for comparisons each treatment: **** indicates $P < 0.0001$; Autophagy, ***$P = 0.002$ for DMSO (red) vs 0.3 μM JQ1 (green) for 24 hours, ***$P = 0.001$ for DMSO (red) vs 0.3 μM JQ1 (green) for 48 hours; Catabolism, ****$P < 0.0001$ for DMSO (red) vs 0.5 μM JQ1 (green) for 24 hours, ****$P < 0.0001$ for DMSO (red) vs 0.3 μM JQ1 (green) for 48 hours.

Supplemental Figure 7.

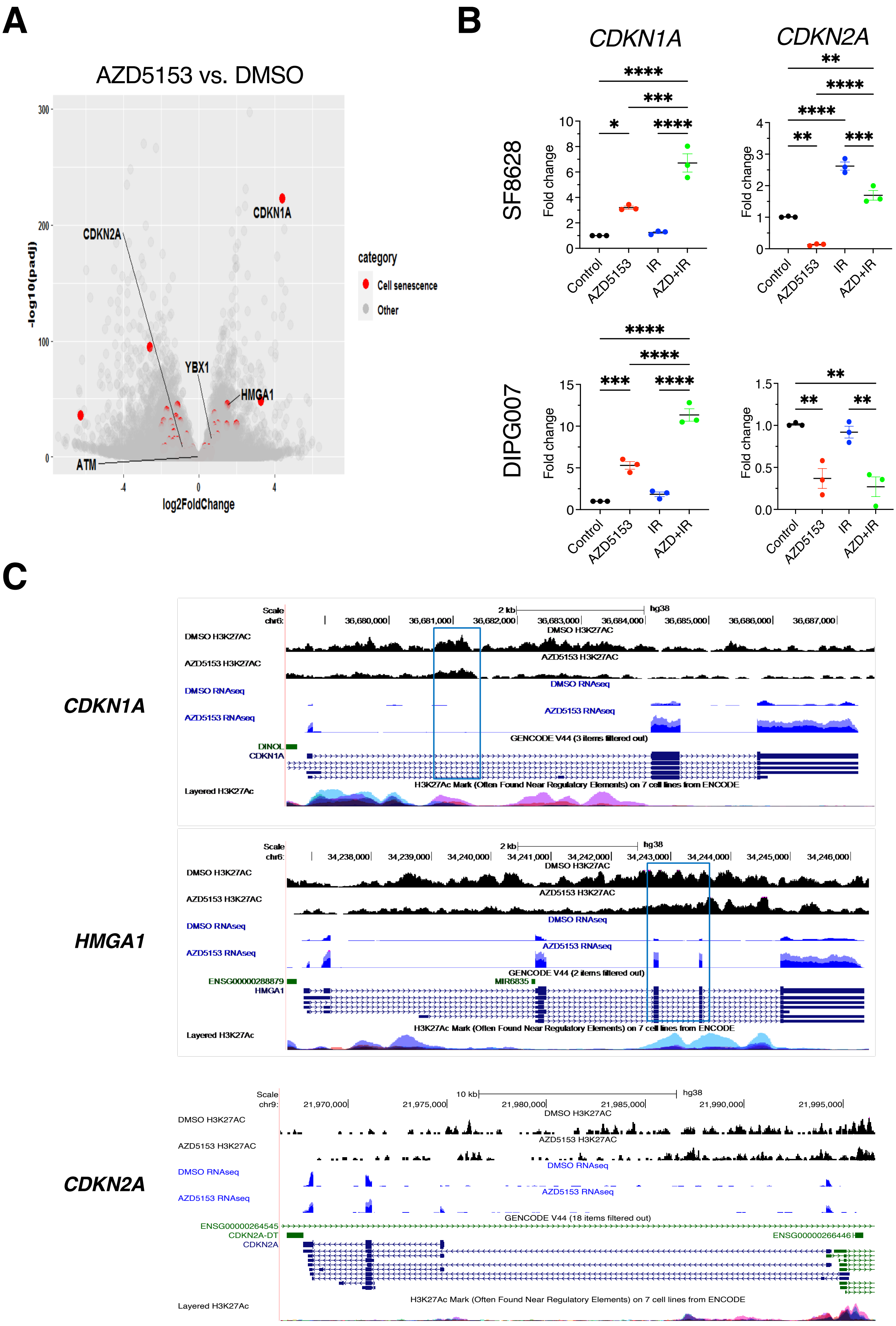

Supplemental Figure 7. Effects of AZD5153 BET bromodomain inhibitor on senescence-associated gene expression in DMG cell. (**a**) Volcano plot of SF8628 DMG cells treated with 0.5 μM AZD5153 or 0.5% DMSO. DMSO-treated samples and AZD5153 treated samples are shown as colored dots and colored by associated pathways (x-axis: $\log_2$ Fold Change; y-axis: $-\log_{10}$ padj values). (**b**) Quantitative RT-PCRs (qRT-PCRs) showing the expression of senescence-associated genes, *CDKN1A* and *CDKN2A,* in irradiated DMG cells (SF8628 and DIPG007) with and without 500 nM AZD5153 or 0.5% DMSO treatment followed by 4Gy IR. Values shown are based on average (mean ± SEM) from triplicate samples. One-way ANOVA with comparison test values between each treatment (n = 3): **** indicates $P < 0.0001$; SF8628, control vs. AZD5153, *$P = 0.0120$ (*CDKN1A*); AZD5153 vs IR, *$P = 0.0226$ (*CDKN1A*); AZD5153 vs. AZD5153 + IR, ***$P = 0.0006$ (*CDKN1A*); control vs. AZD5153, **$P = 0.0011$ (*CDKN2A*); control vs. AZD5153 + IR, **$P = 0.0058$ (*CDKN2A*); IR vs. AZD5153 + IR, ***$P = 0.0008$ (*CDKN2A*); DIPG007, control vs. AZD5153, ***$P = 0.0007$ (*CDKN1A*); AZD5153 vs. IR, **$P = 0.0030$ (*CDKN1A*); control vs. AZD5153, **$P = 0.0044$ (*CDKN2A*); control vs. AZD5153 + IR, **$P = 0.0018$ (*CDKN2A*); AZD5153 vs. IR, *$P = 0.0109$ (*CDKN2A*); IR vs. AZD5153 + IR, **$P = 0.0041$ (*CDKN2A*). (**c**) Gene annotation tracks showing H3K27ac occupancy (black) and gene expression (blue) for *CDKN1A* (upper), *HMGA1* (Middle), and *CDKN2A* (lower) locus.

Supplemental Figure 8.

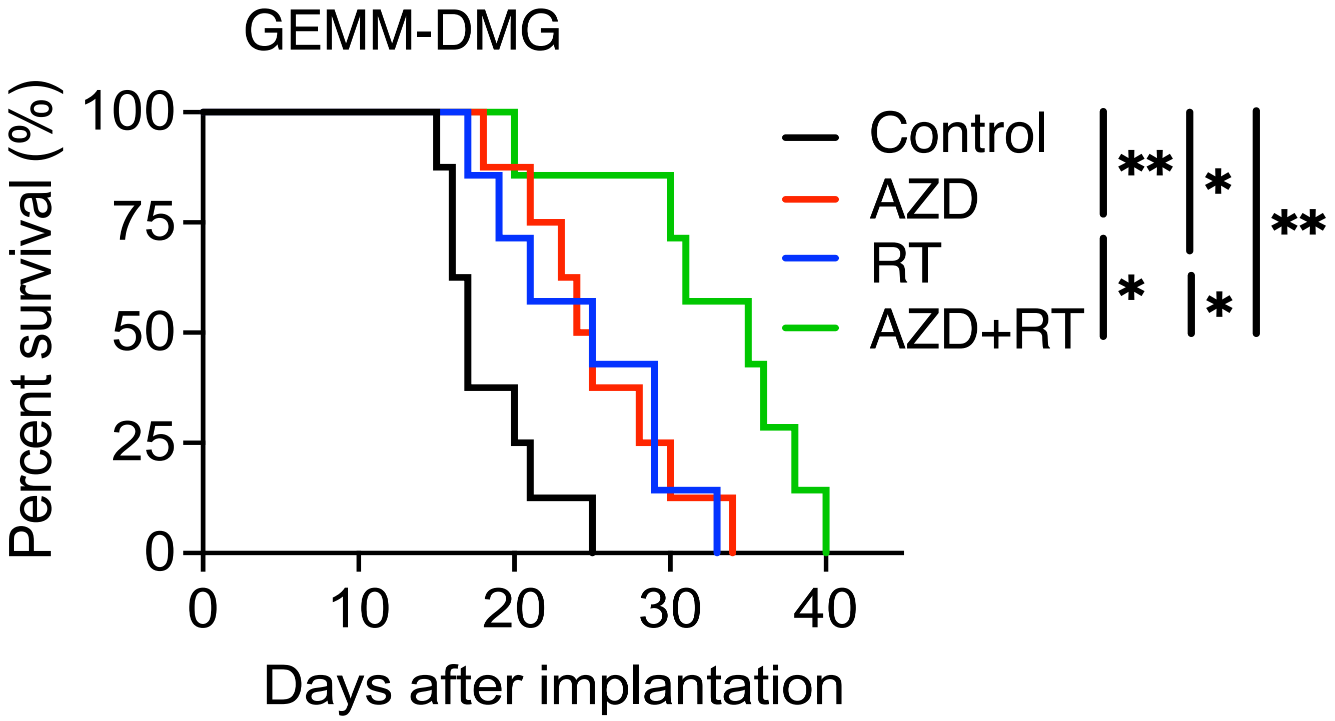

Supplemental Figure 8. BET bromodomain inhibitor enhanced radiation anti-tumor activity in murine DMG model. Mice with GEMM-DMG intracranial tumor were randomized to four treatment groups: control (DMSO, n=8), AZD5153 alone (50mg/kg/day for 5 days a week for two consecutive weeks, n=7), radiation therapy (RT) alone (1.5 Gy, 3 times a week for two consecutive weeks for a total dose of 9 Gy, n=8), and RT + AZD5153 (n=7). Survival plots of each treatment group. Statistical analysis was performed using a log-rank test: control vs. AZD, $^{**}P = 0.008$; control vs. RT, $^{*}P = 0.021$; control vs. AZD + RT, $^{**}P = 0.002$; AZD vs. AZD + RT, $^{*}P = 0.011$; RT vs. AZD + RT, $^{*}P = 0.012$.

Supplemental Figure 9.

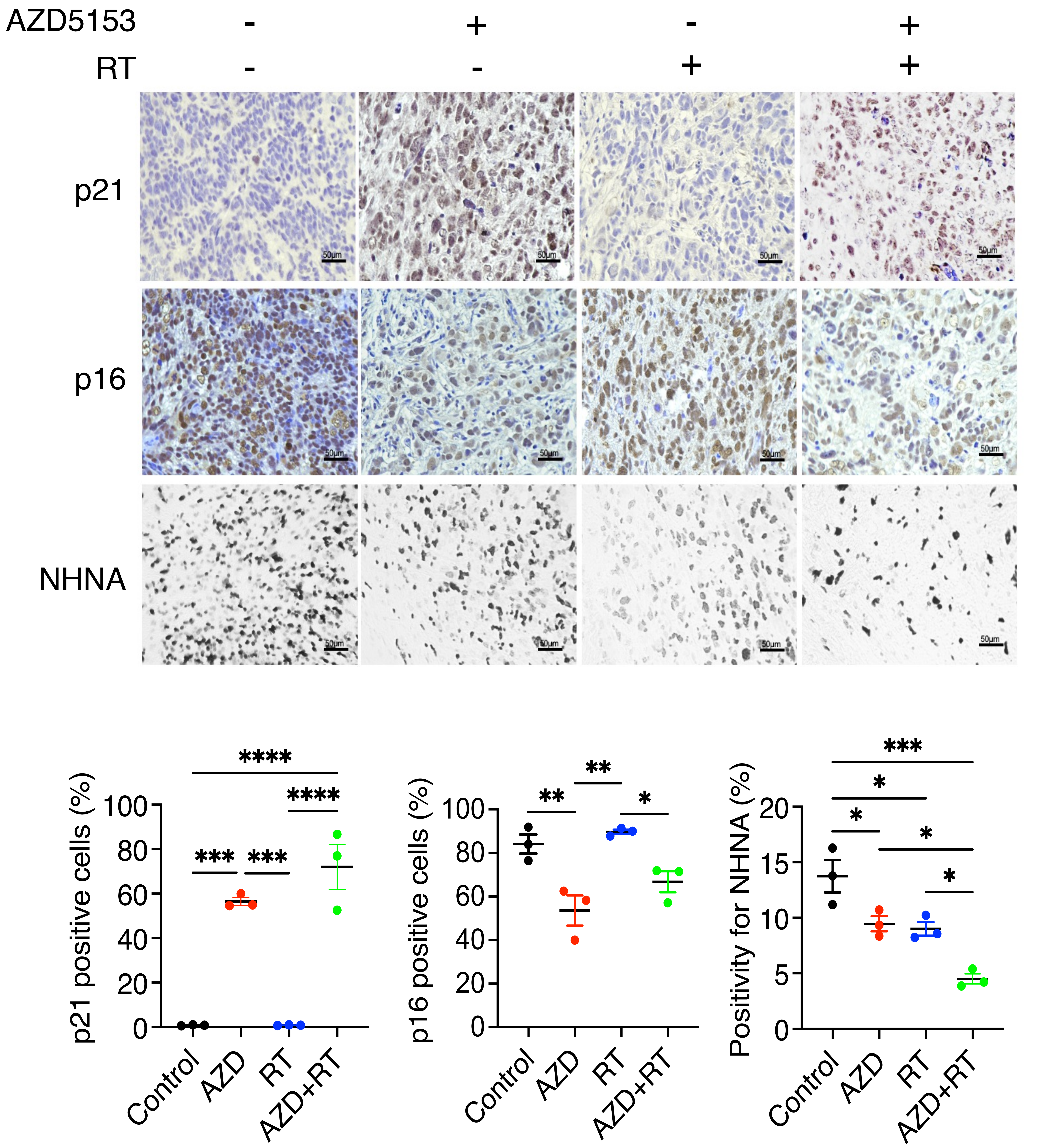

Supplemental Figure 9. Effects of BET bromodomain inhibition on senescence and invasion of tumor cells in vivo. Senescence markers, p21 and p16, were evaluated with the percentage of positive cells was quantified in the different experimental groups by cell counting. Normal human nuclear antigen (NHNA) was evaluated with the cell stain positivity at the tumor invasion area of pons. Magnification used was 400 × (10 × 40). Values (mean ± SEM) representing the average of positive cells in four high-powered fields in three tumor samples (n=3). One-way ANOVA with comparison test values between each treatment: **** indicates $P < 0.0001$; p21, control vs. AZD5153, ***$P = 0.0003$; AZD5153 vs RT, ***$P = 0.0003$; p16, control vs. AZD5153, **$P = 0.0087$; AZD5153 vs. RT, **$P = 0.0031$; RT vs. AZD5153 + RT, *$P = 0.0392$; NHNA, control vs. AZD5153, *$P = 0.0390$; control vs. RT, *$P = 0.0237$; control vs. AZD5153 + RT, ***$P = 0.0004$; AZD5153 vs. AZD5153 + RT, *$P = 0.0185$; RT vs. AZD5153 + RT, *$P = 0.0302$.
