# Supplementary figures and images for: BET bromodomain inhibition potentiates radiosensitivity in models of H3K27-altered diffuse midline glioma
Source: J Clin Invest. 2024 May 21;134(13):e174794. doi: 10.1172/JCI174794 (PMC11213469; doi:10.1172/JCI174794)

### Figure 2A.

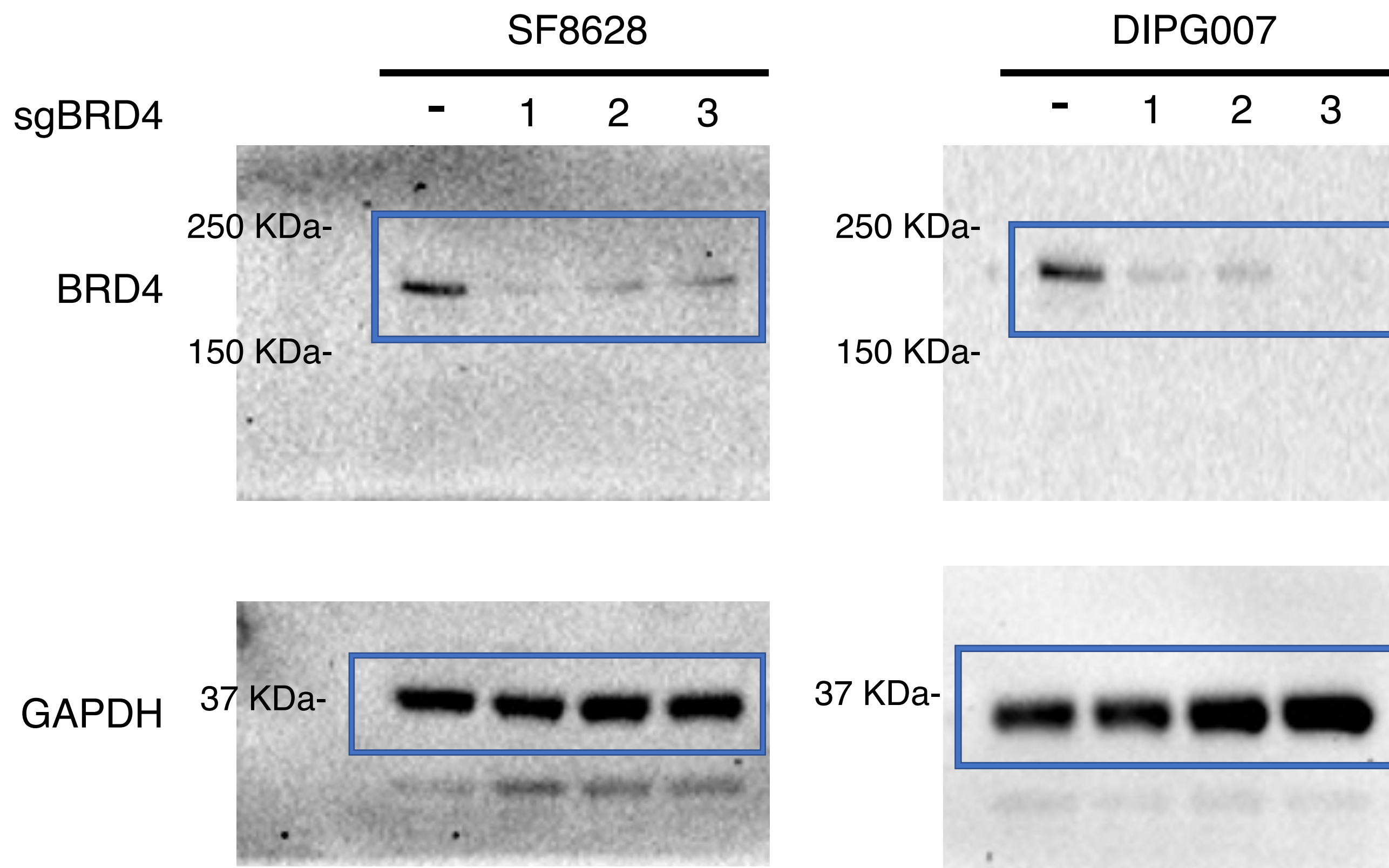

Figure 9A.

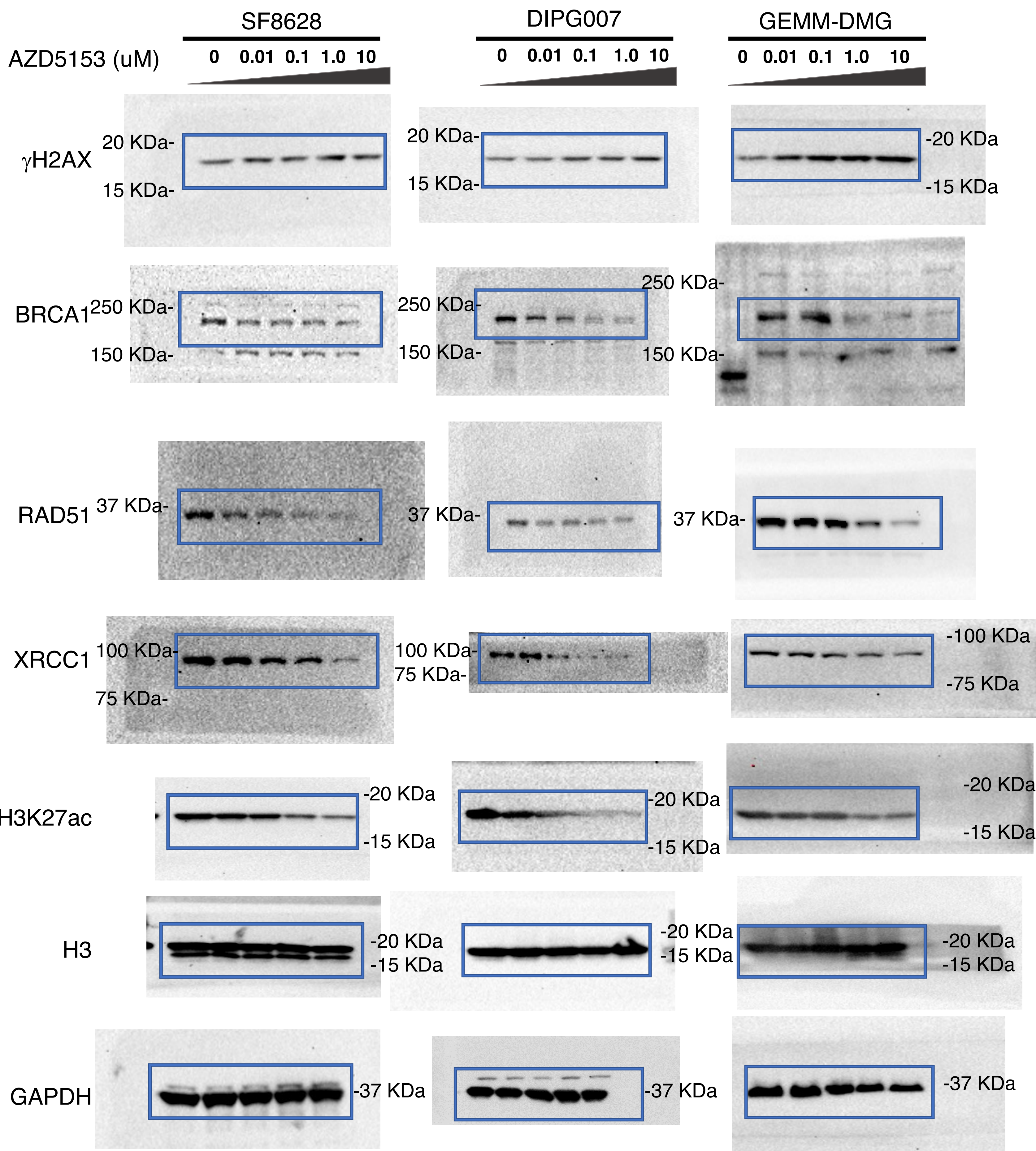

### Figure 9B.

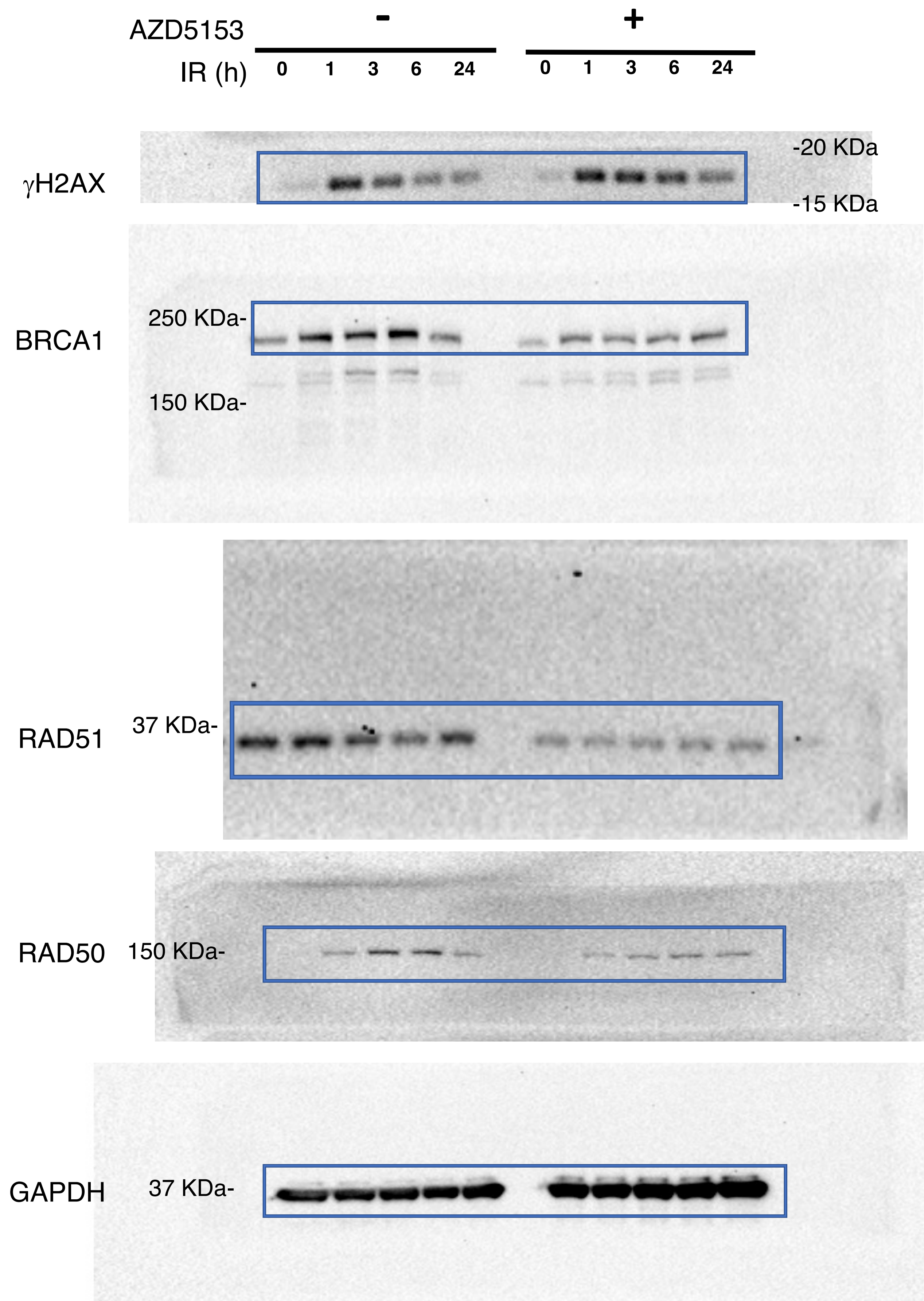

Supplemental Figure 2A.

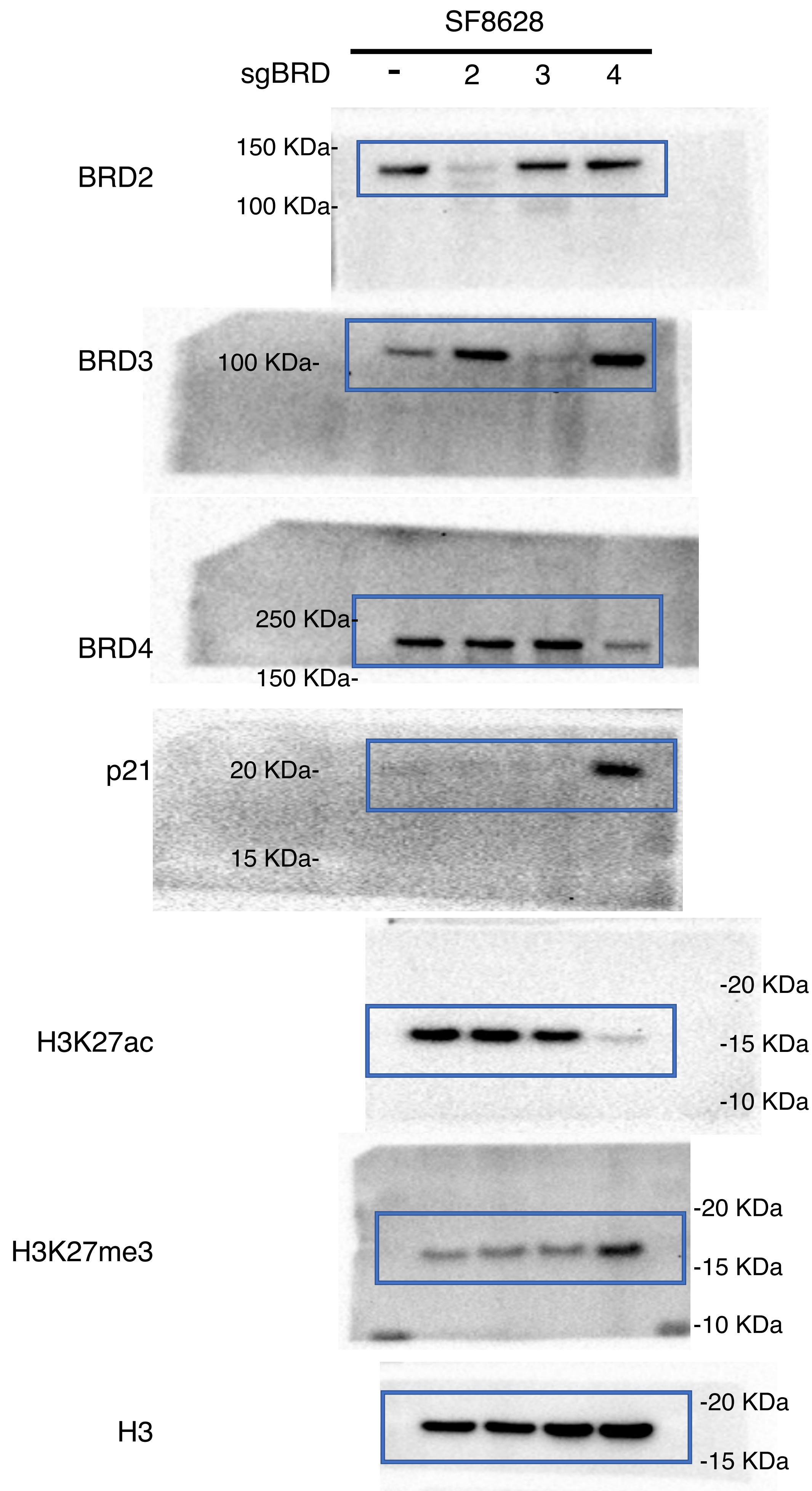

# Supplemental Figure 3A.

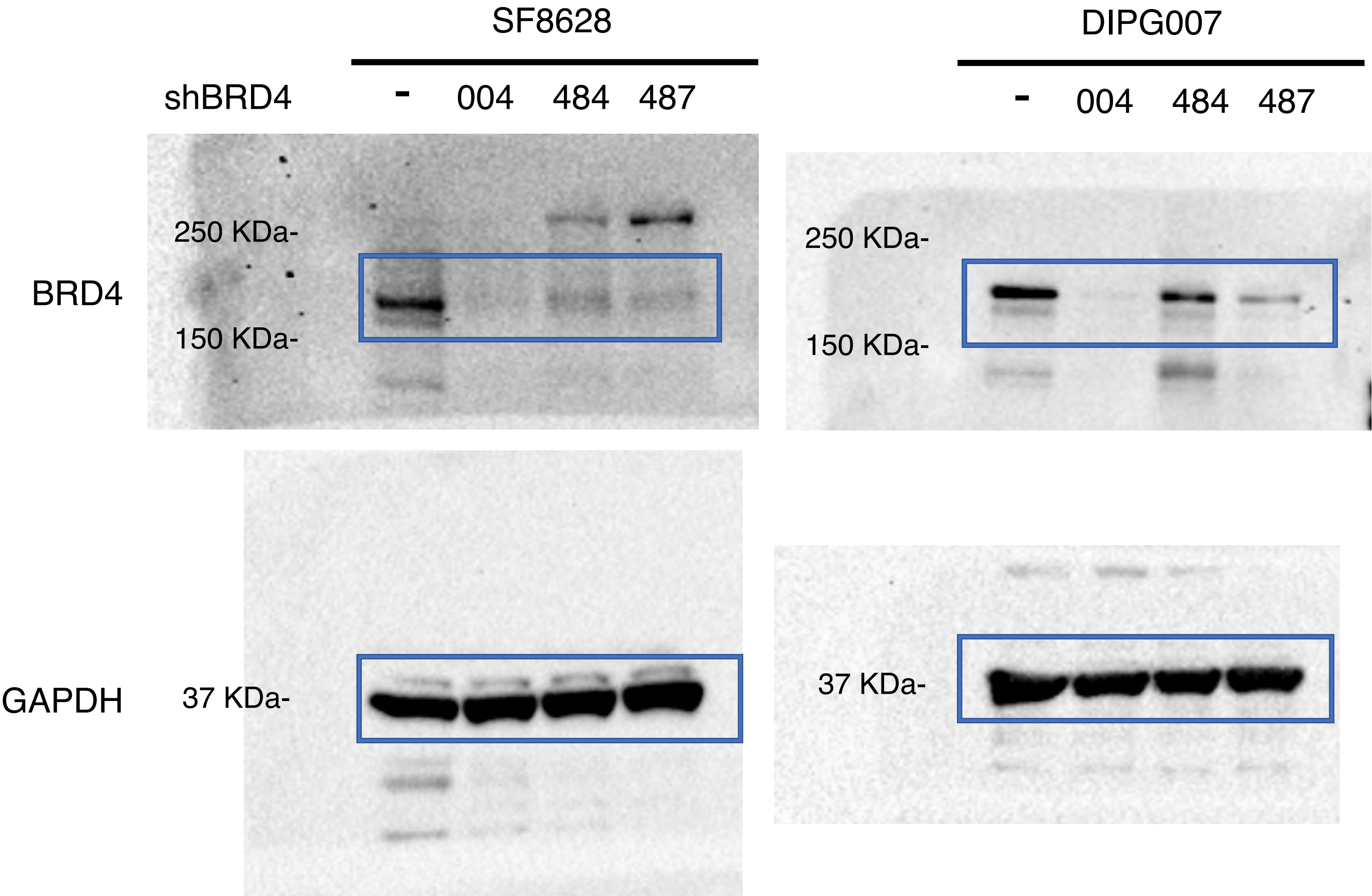

Supplemental Figure 4D.

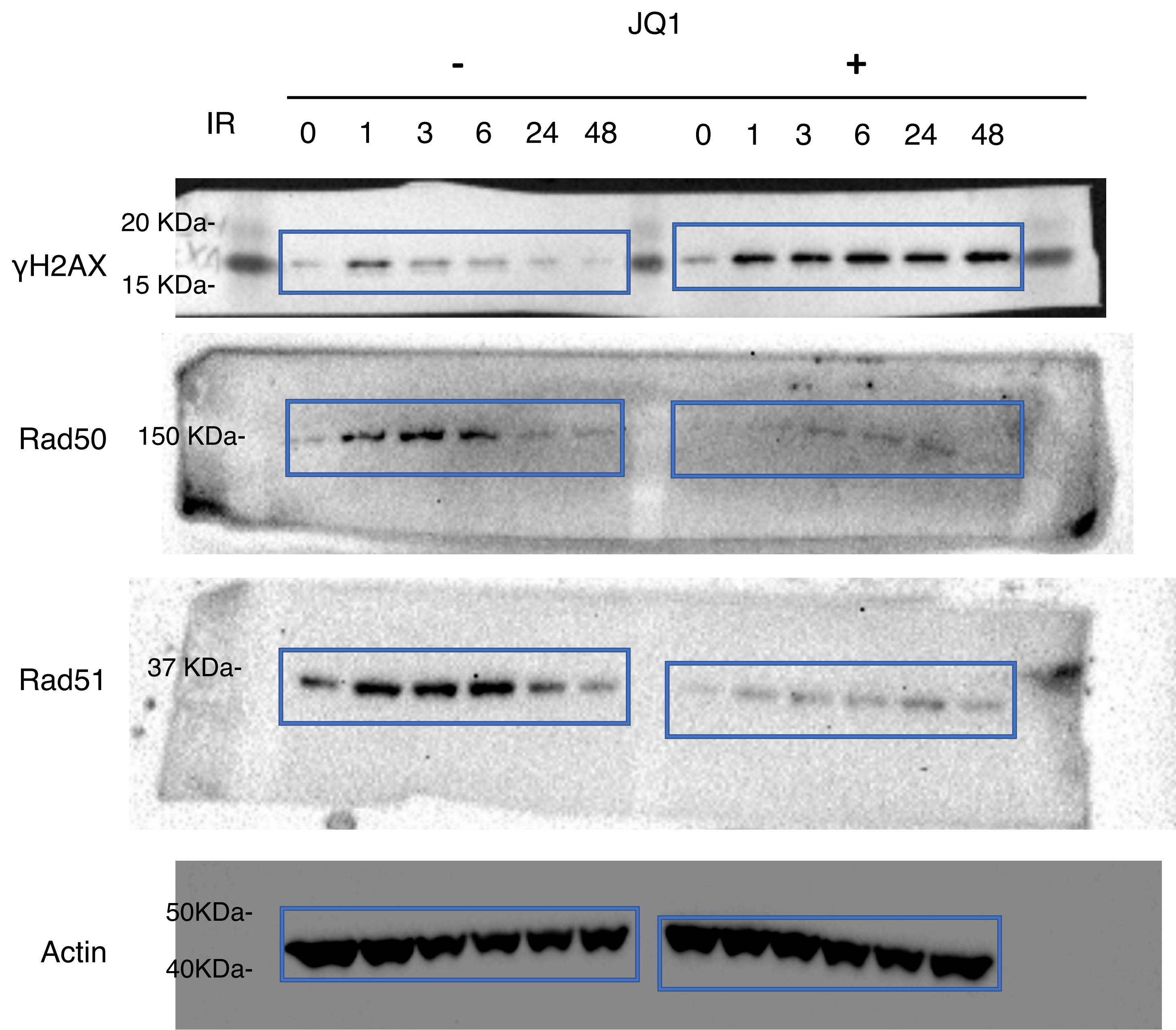

Supplement: Unedited blot and gel images [file jci-134-174794-s206.pdf]
